# No evidence for enzootic plague within black‐tailed prairie dog (*Cynomys ludovicianus*) populations

**DOI:** 10.1111/1749-4877.12546

**Published:** 2021-05-31

**Authors:** Rebecca E. COLMAN, R. Jory BRINKERHOFF, Joseph D. BUSCH, Chris RAY, Adina DOYLE, Jason W. SAHL, Paul KEIM, Sharon K. COLLINGE, David M. WAGNER

**Affiliations:** ^1^ Pathogen and Microbiome Institute Northern Arizona University Flagstaff Arizona USA; ^2^ Environmental Studies Program University of Colorado at Boulder Boulder Colorado USA

**Keywords:** black‐tailed prairie dog, *Cynomys ludovicianus*, plague, prairie dogs, *Yersinia pestis*

## Abstract

*Yersinia pestis*, causative agent of plague, occurs throughout the western United States in rodent populations and periodically causes epizootics in susceptible species, including black‐tailed prairie dogs (*Cynomys ludovicianus*). How *Y. pestis* persists long‐term in the environment between these epizootics is poorly understood but multiple mechanisms have been proposed, including, among others, a separate enzootic transmission cycle that maintains *Y. pestis* without involvement of epizootic hosts and persistence of *Y. pestis* within epizootic host populations without causing high mortality within those populations. We live‐trapped and collected fleas from black‐tailed prairie dogs and other mammal species from sites with and without black‐tailed prairie dogs in 2004 and 2005 and tested all fleas for presence of *Y. pestis*. *Y. pestis* was not detected in 2126 fleas collected in 2004 but was detected in 294 fleas collected from multiple sites in 2005, before and during a widespread epizootic that drastically reduced black‐tailed prairie dog populations in the affected colonies. Temporal and spatial patterns of *Y. pestis* occurrence in fleas and genotyping of *Y. pestis* present in some infected fleas suggest *Y. pestis* was introduced multiple times from sources outside the study area and once introduced, was dispersed between several sites. We conclude *Y. pestis* likely was not present in these black‐tailed prairie dog colonies prior to epizootic activity in these colonies. Although we did not identify likely enzootic hosts, we found evidence that deer mice (*Peromyscus maniculatus*) may serve as bridging hosts for *Y. pestis* between unknown enzootic hosts and black‐tailed prairie dogs.

## Graphical abstract

We studied black‐tailed prairie dog (*Cynomys ludovicianus*) populations in western North America before and during an outbreak of plague (*Yersinia pestis*). *Y. pestis* was not present in the black‐tailed prairie dog populations prior to the onset of the outbreak. Plague activity occurred first in other small mammal species and then in black‐tailed prairie dogs. The outbreak was caused by multiple, distinct lineages of *Y. pestis*.

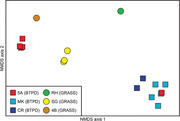

## INTRODUCTION

Plague, caused by the bacterium *Yersinia pestis*, is a zoonotic disease that is ecologically established in rodent foci worldwide, including throughout the western United States (Barnes 1993; Cully & Williams 2001). *Y. pestis* is an obligate pathogen with a natural lifecycle consisting of continued transmission between rodent hosts and flea vectors, with humans representing incidental hosts. Over 350 mammalian species worldwide have been documented to be susceptible to infection with *Y. pestis* (Mahmoudi *et al*. 2020), and in North America alone, >25 flea species have been implicated as vectors (Eisen *et al*. 2009).

A hallmark of plague is its ability to cause epizootics among rodents and epidemics among humans (Cui *et al*. 2013). It is during epizootics that plague can spread rapidly in rodent populations and consequently, humans are most at risk for infection (Gage & Kosoy 2005). In North America, much of the research on plague in native rodent species has been focused on epizootic events in specific mammal species, such as prairie dogs (5 species of colonial ground squirrels in the genus *Cynomys*) and other ground squirrels. In large part this is because these species are diurnal and can live in dense colonies, thereby making them conspicuous, which, in turn, makes it more obvious when plague is reducing their local populations (Eskey & Haas 1940).

How *Y. pestis* persists long‐term in the environment during apparent quiescent periods between rodent epizootics is poorly understood but multiple mechanisms have been proposed, which are not mutually exclusive (not all of these possible mechanisms are discussed here—see Eisen & Gage 2009 for an in depth review). Several laboratory studies have demonstrated the ability of *Y. pestis* to survive in soil under specific conditions (Drancourt *et al*. 2006; Ayyadurai *et al*. 2008; Eisen *et al*. 2008b; Eisen & Gage 2009) and one recent laboratory study documented that *Y. pestis* can survive and replicate within certain amoeba, providing a potential mechanism for its persistence in soil (Markman *et al*. 2018). However, it is not clear that this apparent ability to survive in soil for short periods of time is important to the long‐term persistence of *Y. pestis*; rather, it may just be a vestigial capability leftover from its ancestor, the enteric pathogen *Y. pseudotuberculosis*, which can readily persist in soil. It also has been suggested that the same host and flea species involved in North American plague epizootics may be maintaining *Y. pestis* during enzootic periods between epizootics, albeit with greatly reduced transmission rates (Gage & Kosoy 2005). Under this scenario, epizootic events are triggered in these same host species by certain biological factors, such as the local density of the host species (Davis *et al*. 2004), climate factors (Parmenter *et al*. 1999; Enscore *et al*. 2002; Stenseth *et al*. 2006), or a combination of these and other factors (Biggins & Eads 2019). It also has been proposed that in some foci, there may be two distinct transmission cycles, epizootic and enzootic, that involve different rodent and flea species. This concept purports that the rodent hosts involved in the enzootic cycle (often termed reservoir hosts) experience little obvious mortality, perhaps due to resistance to plague (Tollenaere *et al*. 2010; Andrianaivoarimanana *et al*. 2013) or high reproductive rates that compensate for mortality caused by plague (Gage & Kosoy 2005). In this scenario, long‐term maintenance of *Y. pestis* occurs in the enzootic transmission cycle, and the epizootic transmission cycle occurs only when there is transfer of *Y. pestis* to epizootic hosts from the enzootic cycle, likely via *Y. pestis* infected fleas switching hosts. The idea of separate epizootic and enzootic cycles is appealing in the areas of western North America where prairie dogs, especially black‐tailed prairie dogs (*Cynomys ludovicianus*) and Gunnison's prairie dogs (*C. gunnisoni*), are some of the primary rodent species involved in local epizootics of plague as their local populations can often be reduced by 95–100% following these events (Ecke & Johnson 1952; Lechleitner *et al*. 1968; Barnes 1993; Cully & Williams 2001; Salkeld *et al*. 2016), making it unlikely that these species are also the long‐term reservoir hosts for plague in those areas. Several small rodent species, such as grasshopper mice (*Onychomys* spp.) (Stapp *et al*. 2008, 2009; Kraft & Stapp 2013) and species of *Peromyscus* (deer mice) and *Microtus* (voles) (Bacon & Drake 1958; Kartman *et al*. 1958; Quan & Kartman 1962; Goldenberg *et al*. 1964; Poland & Barnes 1979; Poland *et al*. 1994; Perry & Fetherston 1997; Gage & Kosoy 2005) have been postulated to serve as plague reservoirs in North America, but data directly linking these possible enzootic hosts to epizootic events in other rodent species are lacking (Gage & Kosoy 2005; Eisen & Gage 2009).

To examine plague transmission between and/or within possible enzootic and epizootic hosts, we examined fleas collected across 2 years from rodents and other mammals on and off black‐tailed prairie dog colonies in the state of Colorado in the United States (off colony sites are hereafter referred to as grassland sites and black‐tailed prairie dog colonies as prairie dog colonies or sites). The aims of our study were to: (1) test for the presence of *Y. pestis* in fleas collected from mammals trapped at both grassland sites and prairie dog colonies; (2) examine temporal patterns of *Y. pestis* occurrence at grassland sites and prairie dog colonies; and (3) use genotyping of *Y. pestis* DNA present in individual infected fleas to reconstruct the genetic population structure of *Y. pestis* across this landscape.

## MATERIALS AND METHODS

### Collection sites

**Figure 1:**
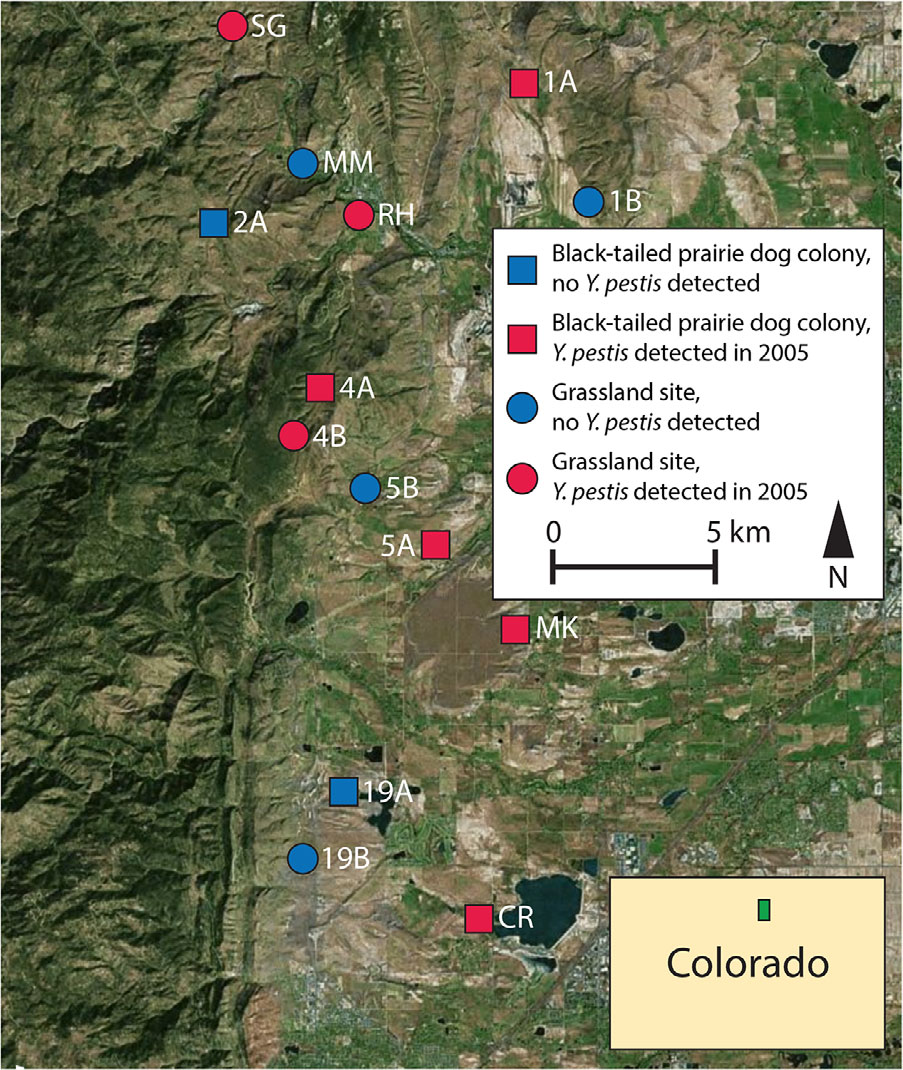
Location of 14 black‐tailed prairie dog colonies and grassland sites in Boulder County, Colorado where fleas were collected from black‐tailed prairie dogs and small mammals in 2004 and/or 2005. Site abbreviations for sites added in 2005 start with a letter instead of a numeral. Circles and squares represent grassland sites and black‐tailed prairie dog colonies, respectively, and the colors of these shapes indicate presence (red) or absence (blue) of *Y. pestis* in individual fleas collected from these sites in 2005. The inset indicates the location of the study area within the state of Colorado in the United States.

Fleas were collected from live mammals at 2 types of sites in Boulder County, Colorado, USA, in 2004 and 2005: black‐tailed prairie dog colonies and grassland sites without black‐tailed prairie dogs present (Fig. 1; Table 1). We examined 9 sites (5 black‐tailed prairie dog colonies and 4 grassland sites) across both years, including 2004 in which no *Y. pestis* was detected at any of the sites and 2005 in which plague was widespread across our study area (Fig. 1). Because the occurrence of plague at any particular location in the western United States is almost impossible to predict in any given year, this outcome was fortuitous and allowed us the rare opportunity to both compare rodent and flea populations at the same sites with and without plague present, and also to collect and examine fleas and mammals during the course of an active plague outbreak. To better characterize plague activity in the overall region after it was first detected in June 2005, the study was expanded in 2005 to include 5 additional sites where plague was suspected (2 additional black‐tailed prairie dog colonies and 3 additional grassland sites; see Fig. 1, wherein site abbreviations for additional sites added in 2005 start with a letter instead of a numeral), for a total of 14 sites sampled in 2005 (June–September). Prior to the 2005 outbreak described here, the last known plague activity among black‐tailed prairie dogs in this region occurred in 2000, which affected only a small number of colonies, with the last major epizootic in 1994 (Collinge & Ray 2006). All sites were located in grasslands near the transition between the Great Plains ecoregion and the Rocky Mountains; the elevational range among the sites was 1630–1920 m.

**Table 1:** Site information, rodent and flea species collected at each site, and *Y. pestis* presence in fleas

Site	Site type^†^	Plague positive in 2005	Range of 2004 flea collection dates	*Y. pestis* positive fleas/total fleas in 2004	Mammal hosts trapped in 2004‡(#/species)	Flea species collected from hosts in 2004^§^(#/species)	Range of 2005 flea collection dates	*Y. pestis* positive fleas/total fleas (%) in 2005	Collection date of first *Y. pestis* positive flea in 2005	Mammal hosts trapped in 2005^‡^(#/species)	Flea species collected in 2005^§^(#/species)
4B	GRASS	Yes	25 Aug–26 Aug	0/9	***M. ochrogaster* (7)** *P. maniculatus* (20)	***A. wagneri* (8)** ***O. leucopus* (4)**	30 May–19 Aug	2/24 (8.3)	1‐Jun	***M. ochrogaster* (2)** ^+^ *P. maniculatus* (5)	^+^ *A. wagneri* (6) ***C. pseudagyrtes* (11)** *O. leucopus* (7)
RH	GRASS	Yes	—	—			06 Jun–10 Jun	3/18 (16.7)	9‐Jun	^+^ ***P. maniculatus* (8)**	^+^ ***A. wagneri* (16)** *M. telchinum* (2)
SG	GRASS	Yes	—	—			20 Jun–24 Jun	43/58 (74.1)	21‐Jun	^+^ ***M. ochrogaster* (1)**	^+^ ***C. pseudagyrtes* (29)** ^+^ ***O. leucopus* (29)**
1A	BTPD	Yes	28 Jun–02 Sep	0/215	***C. ludovicianus* (58)** *P. maniculatus* (52) *S. tridecemlineatus* (2)	*A. wagneri* (24) *M. telchinum* (7) ***O. hirsuta* (108)** *P. simulans* (1) *T. fotus* (2)	06 Jun–02 Sep	2/248 (0.8)	27‐Jun	^+^ ***C. ludovicianus* (25)** ^+^ *P. maniculatus* (46)	^+^ *A. wagneri* (31) *E. wenmanni* (2) *M. telchinum* (22) ^+^ ***O. hirsuta* (192)** *O. leucopus* (1)
5A	BTPD	Yes	26 Jul–24 Aug	0 / 328	***C. ludovicianus* (22)** *P. maniculatus* (49) *C. hispidus* (3)	*A. wagneri* (27) *M. telchinum* (2) ***O. hirsuta* (41)**	30 May–19 Aug	20/158 (12.7)	26‐Jul	^+^ ***C. ludovicianus* (19)** *P. maniculatus* (55) ^+^ *S. audobonii* (2)	*A. wagneri* (45) *C. inaequalis* (5) ^+^ *E. glacialis* (7) *M. telchinum* (2) ^+^ ***O. hirsuta* (99)**
4A	BTPD	Yes	26 Jul–26 Aug	0/120	*C. hispidus* (1) ***C. ludovicianus* (9)** *P. maniculatus* (40)	***A. wagneri* (23)** *M. telchinum* (6) *O. hirsuta* (17)	30 May–19 Aug	1/76 (1.3)	28‐Jul	^+^ ***C. ludovicianus* (4)** ***P. maniculatus* (18)**	*A. wagneri* (23) *M. telchinum* (15) ^+^ ***O. hirsuta* (38)**
MK	BTPD	Yes	—	—			15 Aug–02 Sep	215/332 (64.5)	24‐Aug	^+^ ***C. ludovicianus* (5)** ^+^ *P. maniculatus* (98) *S. audobonii* (1)	^+^ *A. wagneri* (107) *C. inaequalis* (4) *M. telchinum* (4) ^+^ ***O. hirsuta* (220)**
CR	BTPD	Yes	—	—			28 Sep	8/17 (47.1)	28‐Sept	^+^SWAB^¶^	*E. glacialis* (1) ^+^ ***O. hirsuta* (16)**
MM	GRASS	No	—	—			03 Jun–08 Jun	0/1 (0)	NA	***P. maniculatus* (1)**	***A. wagneri* (1)**
1B	GRASS	No	31 Aug–03 Sep	0/59	*C. hispidus* (21) ***M. ochrogaster* (6)** ***P. maniculatus* (31)** *R. megalotis* (9)	***A. wagneri* (38)** *M. telchinum* (3) *O. leucopus* (14)	06 Jun–02 Sep	0/47 (0)	NA	*C. hispidus* (12) *M. ochrogaster* (6) *M. pennsylvanicus* (1) ***P. maniculatus* (26)**	***A. wagneri* (28)** *M. telchinum* (5) *O. leucopus* (14)
2A	BTPD	No	28 Jun–09 Sep	0/844	***C. ludovicianus* (46)** *P. maniculatus* (54)	*A. wagneri* (61) *M. telchinum* (18) ***O. hirsuta* (121)**	13 Jun–09 Sep	0/36 (0)	NA	***C. ludovicianus* (25)**	***O. hirsuta* (36)**
5B	GRASS	No	24 Aug–27 Aug	0/22	*M. ochrogaster* (5) *N. mexicana* (11) ***P. maniculatus* (15)**	*A. wagneri* (2) *A. nudatus* (1) *M. telchinum* (1) ***O. leucopus* (31)**	30 May–19 Aug	0/12 (0)	NA	*M. ochrogaster* (2) *M. pennsylvanicus* (2) ***P. maniculatus* (8)**	***A. wagneri* (4)** *C. pseudagyrtes* (3) *M. telchinum* (1) ***O. leucopus* (4)**
19A	BTPD	No	12 Jul–09 Sep	0/528	***C. ludovicianus* (83)** *P. maniculatus* (52)	*A. wagneri* (81) *M. telchinum* (13) ***O. hirsuta* (209)**	13 Jun–09 Sep	0/86 (0)	NA	***C. ludovicianus* (57)** *M. ochrogaster* (6) *P. maniculatus* (61) *S. audobonii* (1)	*A. wagneri* (10) *C. pseudagyrtes* (2) *E. wenmanni* (1) *E. glacialis* (1) *M. telchinum* (22) *O. leucopus* (12) ***O. hirsuta* (38)**
19B	GRASS	No	18 Jun	0/1	***P. maniculatus* (28)**	***M. telchinum* (2)**	13 Jun–09 Sep	0/10 (0)	NA	***P. maniculatus* (9)**	*C. pseudagyrtes* (1) ***M. telchinum* (9)**
Total	—	—		0/2126	8 species	7 species	—	294/1123 (26.2)	—	6 species	8 species

^†^ GRASS, grassland sites; BTPD, black‐tailed prairie dog colonies. ‡ The rodent species that yielded the most collected fleas from each site is in bold; full species names are: *Chaetodipus hispidus*, *Cynomys ludovicianus*, *Microtus ochrogaster*, *Microtus pennsylvanicus*, *Neotoma mexicana*, *Peromyscus maniculatus*, *Reithrodontomys megalotis*, *Spermophilus tridecemlineatus*, and *Sylvilagus audobonii*. § The most common flea species collected from each site is in bold; full species names are: *Aetheca wagneri*, *Anomiopsyllus nudatus*, *Cediopsylla inaequalis*, *Ctenophthalmus pseudagyrtes*, *Epitedia wenmanni*, *Euhoplopsyllus glacialis*, *Malareus telchinum*, *Orchopeas leucopus*, *Oropsylla hirsuta*, *Pulex simulans*, and *Thrassis fotus*. ¶ SWAB = Collected from a vacant prairie dog burrow. + Indicates a *Y. pestis* positive flea species or a rodent species that was carrying a *Y. pestis* positive flea.

### Host and flea collection

**Figure 2:**
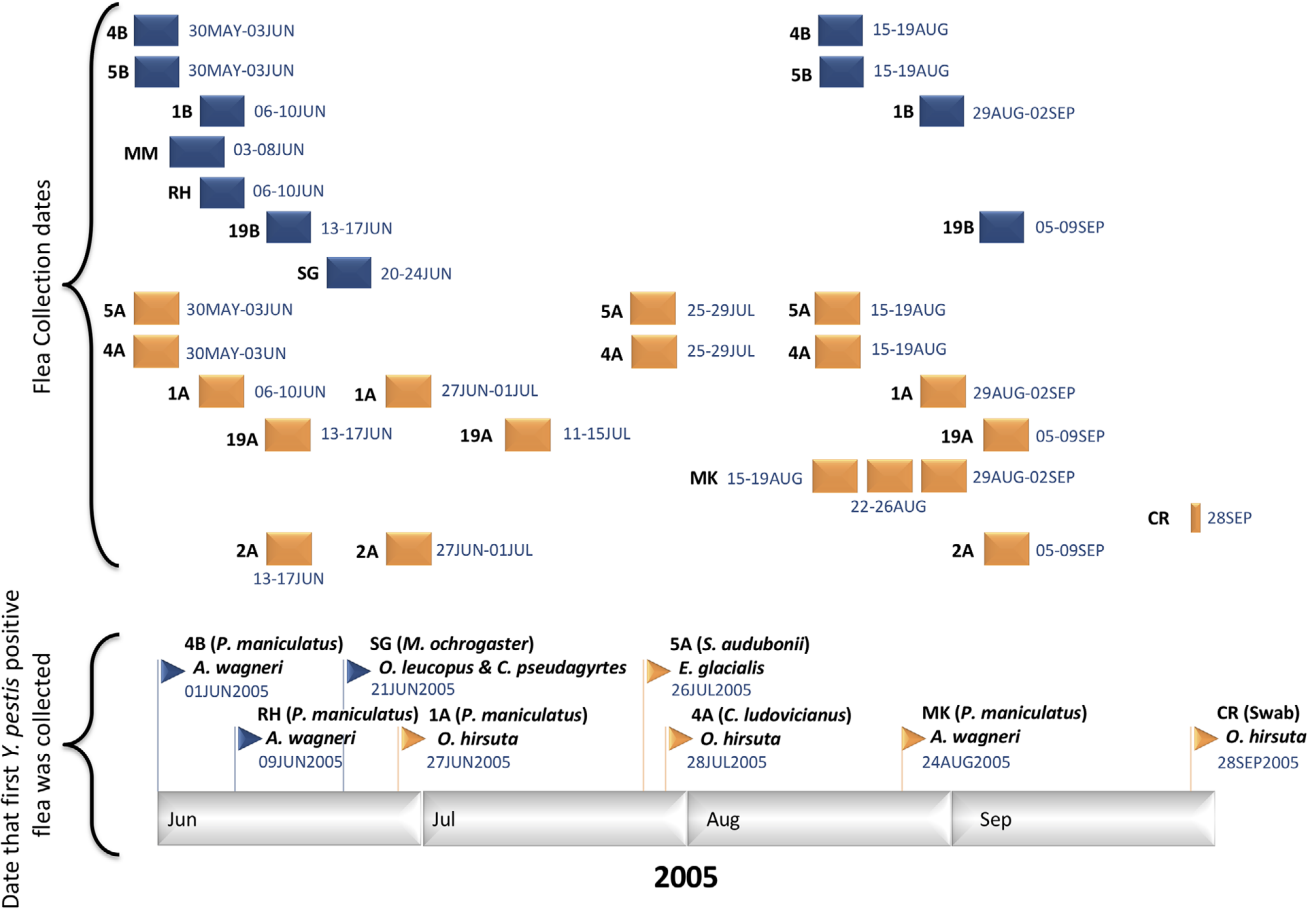
Top: Specific dates in 2005 when black‐tailed prairie dogs and/or small mammals were trapped at 7 grassland sites (blue; just small mammal trapped) and 7 black‐tailed prairie dog colonies (orange; both black‐tailed prairie dogs and small mammals trapped) and fleas were collected from the trapped mammals. Bottom: Specific date that the first *Y. pestis* positive flea was collected at each of the 8 sites that yielded *Y. pestis* positive fleas. The species of the first *Y. pestis* positive flea from each site, as well as the rodent species that it was collected from (in parentheses), is indicated. “Swab” indicates the first *Y. pestis* positive flea was collected via a burrow swab.

Host sampling and flea collection methods are described in detail elsewhere (Snall *et al*. 2008; Brinkerhoff *et al*. 2008, 2010). Black‐tailed prairie dogs were sampled with 48–50 traps per site during multiple 4‐day sampling session in each year (Fig. 2) using Tomahawk traps (16"L × 5"W × 5"H, Tomahawk Live Trap, Hazelhurst, WI) with 25‐m spacing between individual traps. Small rodents were typically sampled twice per year, once in May/June and once in August/September, also during mostly 4‐day sampling sessions (Fig. 2), using the same sampling grids used for the prairie dogs on prairie dog colonies and Sherman live‐traps (7.6 × 8.9 × 22.9 cm; H. B. Sherman Traps, Tallahassee, FL). Exceptions were the 2005‐only sites MM, MK, RH, and SG, which were located on private property; these were sampled opportunistically (Fig. 2). Another exception was the 2005‐only site CR, wherein a small number of fleas (*n* = 17) were collected in September 2005 by swabbing prairie dog burrows (Table 1). Traps were pre‐baited with a corn‐oat‐barley mixture for 3 days with the traps held open. After pre‐baiting, traps were re‐baited, set for 3 h per day for 4 consecutive days. Prairie dog traps were set between 0600 and 0800 and checked before 1200, and small rodent traps were set in the evening and checked between 0600 and 0900.

All trapped animals were anesthetized using vaporized Isoflurane (Halocarbon Products Corporation, River Edge, NJ). We used combs (black‐tailed prairie dogs) and toothbrushes (small rodents) to find fleas, which were then collected from the hosts with fine‐tipped forceps. Fleas were also collected with forceps from the anesthetizing chamber and from the white plastic processing animal tray. Collecting fleas from captured animals allowed individual fleas to be definitely associated with a particular animal host. Fleas from each mammal were stored in 2% saline solution with a small amount of Tween 80 (polysorbate) and were later identified to species using taxonomic keys (Hubbard 1968; Furman & Catts 1982). In addition to flea information, mammal species identification, sex, weight, and length measurements were recorded, and blood samples collected. All individuals were marked with uniquely numbered ear tags and all trapping and other animal handling procedures were approved by the University of Colorado IACUC.

### Serological testing of small mammals

Blood samples were collected in the field from a subset of the captured mammals using Nobuto filter paper strips and were kept frozen at −20°C prior to elution for serological screening. Sera were initially tested for antibodies against the *Y. pestis* fraction 1 (F1) antigen using a passive hemagglutination assay (PHA) (Chu 2000); the antigen was produced and purified at the U.S. Centers for Disease Control and Prevention in Fort Collins, Colorado. Samples that tested positive by PHA were confirmed by inhibition assay and samples with titers of at least 1:32 were considered positive.

### DNA extraction/Molecular work

To obtain a definitive match between individual *Y. pestis* positive fleas and specific host individuals and, thereby, identify the particular vector and mammal species combinations that may be important for *Y. pestis* transmission in this system, DNA was extracted from individual fleas using DNeasy blood and tissue extraction kits (Qiagen, Valencia, CA) following an existing protocol (Allender *et al*. 2004). We screened for the presence of *Y. pestis* DNA in all individual flea DNA extracts using 2 real‐time PCR assays (Mitchell *et al*. 2017). One assay targets the plasminogen activator (*pla*) gene on the *Y. pestis* pPCP1 plasmid, whereas the other assay targets the 3a region on the *Y. pestis* chromosome (Radnedge *et al*. 2001). DNA extracts from individual fleas with a concentration of *Y. pestis* DNA sufficient to support additional molecular testing were genetically characterized with multi‐locus variable number‐tandem repeat (VNTR) analysis (MLVA). The *Y. pestis* MLVA system utilized in this study consists of 43 different VNTRs that allow for fine‐scale resolution of more recent phylogenetic relationships that could arise during an epizootic scenario (Girard *et al*. 2004; Vogler *et al*. 2007; Colman *et al*. 2009). To evaluate the specificity of a published nested PCR assay (Hanson *et al*. 2007) that is commonly utilized to screen for the presence of *Y. pestis* DNA in flea DNA extracts but was not utilized in this study, the external (5′‐catccggctcacgttattatggtacc‐3′, 5′‐cttggatgttgagcttcctacag‐3′) and internal (5′‐cacacctaatgccaaagtctttgcgg‐3′, 5′‐cgccaatagagacagaatctccac‐3′) PCR primers for this assay were screened *in silico* across 193 653 bacterial genomes in GenBank using ViPR v1.0 (https://github.com/TGenNorth/vipr). The evaluated genomes included 378 annotated as *Y. pestis*; 306 of these *Y. pestis* genomes contained a mostly full length *pla* gene.

### Statistical analysis

Several factors were examined that could have potentially influenced the presence of *Y. pestis* at a particular site. Chi‐square tests of independence were used to determine if detected *Y. pestis* presence at a site was independent of detected *Y. pestis* presence at the nearest neighboring site, as well as site type (grassland vs. prairie dog colony); Chi‐square tests were conducted using JMP IN 1.04 (SAS Inst., Cary, NC). We used non‐metric multidimensional scaling (NMDS) ordination to assess population structure patterns of *Y. pestis* found in the flea samples (Clarke & Warwick 2001). To serve as a comparison to the NMDS ordination, a midpoint rooted neighbor‐joining tree was built for the samples using PAUP software (Swofford 1993) and a distance matrix generated from amplicon size data for the 43 *Y. pestis* VNTR loci. A Mantel test was performed to examine the relationship between geographic distance and *Y. pestis* genetic distance using GenAlEx 6 (Peakall & Smouse 2006), with the latter distance based upon the MLVA data.

## RESULTS

### Temporal and spatial occurrence of *Y. pestis*

In 2004, a total of 2126 fleas were collected from 684 small mammals (including 218 black‐tailed prairie dogs) trapped at 9 sites (4 grassland sites, 5 prairie dog colonies) and none of the fleas were positive for *Y. pestis* (Table 1). However, *Y. pestis* was detected in fleas collected from 4 of these 9 sites in 2005. *Y. pestis* positive fleas were collected from grassland site 4B on June 1; *Y. pestis* positive fleas were subsequently collected from prairie dog colony 1A at the end of June and prairie dog colonies 4A and 5A in late July (Fig. 2). In total, we collected 697 fleas from 392 small mammals (including 130 prairie dogs) from these 9 sites in 2005; 25 fleas collected from these 9 sites (3.6%) were positive for *Y. pestis* (Table 1).

*Y. pestis* positive fleas were collected from 4 of the 5 sites that were not sampled in 2004 but were opportunistically sampled in 2005. We collected an additional 426 fleas from 114 small mammals (including 5 prairie dogs) captured at these 5 sites, of which 269 fleas (63%) were positive for *Y. pestis* (Table 1). *Y. pestis* positive fleas were collected from grassland sites RH and SG in June, and from prairie dog colonies MK and CR in August and September, respectively (Fig. 2); no *Y. pestis* positive fleas were collected at grassland site MM.

In total, across the 14 combined study sites sampled in 2005, 1123 fleas were collected from 506 small mammals (including 135 black‐tailed prairie dogs). Of those fleas, 294 (26.2%) collected at 3 grassland sites and 5 prairie dog colonies were *Y. pestis* positive (Fig. 1). The 294 *Y. pestis* positive fleas (Table S1, Supporting Information) represented 31.5% of the total number of fleas (*n* = 932) collected from the 8 *Y. pestis*‐positive sites that year.

### Hosts and flea species distribution

In 2004, 7 flea species were collected from 8 host species; in 2005, 8 flea species were collected from 6 host species (Table 1). In general, the specific mammal species captured and the specific flea species collected from those mammal species at each site were consistent across the 2 years (Table 1). The most widespread host species in both years were deer mice, which were trapped at 11 of the 14 sites, including 5 of the 7 prairie dog colonies (Tables 1 and 2). Deer mice also harbored more flea species (*n* = 6) than any other small mammal species examined in this study (Table 2). Four flea species were found at both grassland sites and prairie dog colonies, but the most widespread species in both years (i.e. the species found at the most sites) were *Aetheca wagneri* and *Malareus telchinum* (Table 1); *A. wagneri* from multiple sites were *Y. pestis* positive but all *M. telchinum* were *Y. pestis*‐negative (Tables 1 and 2).

**Table 2:** Host and flea combinations from 2005 associated with plague occurrence in our study area

Host species (all sites where it was captured in 2005^†^)	Host yielded *Y. pestis* positive fleas	All flea species collected from this host species across all sites	*Y. pestis*‐positive/total number of fleas collected from host species	Site type(s) where flea species were collected^†^
***P. maniculatus*** (GRASS: 19B, 1B, **4B**, 5B, MM, **RH**; BTPD: 19A, **1A**,​ 4A, 5A, **MK**)	YES	***A. wagneri***	**9/266**	**GRASS/BTPD**
		***O. hirsuta***	**1/4**	**BTPD**
		*M. telchinum*	0/72	GRASS/BTPD
		*O. leucopus*	0/3	GRASS/BTPD
		*E. wenmanni*	0/2	BTPD
		*C. pseudagyrtes*	0/1	GRASS
***C. ludovicianus*** (BTPD: 19A, **1A**, 2A, **4A**, **5A**, **MK**)	YES	***O. hirsuta***	**232/615**	**BTPD**
***M. ochrogaster*** (GRASS: 1B, 4B, 5B, **SG**;​ BTPD: 19A)	YES	***C. pseudagyrtes***	**20/42**	**GRASS**/BTPD
		***O. leucopus***	**23/60**	**GRASS**/BTPD
		*M. telchinum*	0/6	GRASS/BTPD
		*E. wenmanni*	0/1	BTPD
***S. audobonii*** (BTPD: 19A, **5A**, MK)	YES	***E. glacialis***	**1/8**	**BTPD**
		*C. inaequalis*	0/9	BTPD
		*O. hirsuta*	0/4	BTPD
*M. pennsylvanicus* (GRASS: 1B, 5B)	NO	*O. leucopus*	0/4	GRASS
		*C. pseudagyrtes*	0/3	GRASS
		*A. wagneri*	0/1	GRASS
		*M. telchinum*	0/1	GRASS
*C. hispidus* (GRASS: 1B)	NO	*A. wagneri*	0/2	GRASS
**SWAB** (BTPD: **CR**)	YES	***O. hirsuta***	**8/16**	**BTPD**
		*E. glacialis*	0/1	BTPD

^†^ GRASS, grassland sites; BTPD, black‐tailed prairie dog colonies. Bolded text indicates host/flea combinations that yielded *Y. pestis* positive fleas and the specific site type(s) where *Y. pestis* positive fleas were collected.

### Host‐flea combinations

The vast majority of the 1123 fleas collected in 2005 were obtained from black‐tailed prairie dogs (*n* = 617; 54.9%) or deer mice (*n* = 348; 31.0%; Table 2). Two flea species stood out in the 2005 collections, *Oropsylla hirsuta* and *A. wagneri*, which accounted for 56.9% and 24.1% of the total number of fleas collected, respectively, and 82.0% and 3.1% of the *Y. pestis* positive fleas, respectively (Table 2). As expected, *O. hirsuta*, a prairie dog flea, was only collected from prairie dog colonies and primarily from black‐tailed prairie dogs, and *Y. pestis*‐positive individuals of this species occurred at all 5 prairie dog sites that experienced plague in 2005 (Tables 1 and 2). However, within prairie dog sites *O. hirsuta* was also collected from other rodent species, including deer mice (*n* = 4) and desert cottontail rabbits (*Sylvilagus audubonii*, *n* = 4); *Y. pestis* positive *O. hirsuta* were collected from black‐tailed prairie dogs (*n* = 234) and swabs of prairie dog burrows (*n* = 8; Table 2). All of the *Y. pestis* positive and a majority of the total collected *A. wagneri* (98.9%) were obtained from deer mice, but *Y. pestis* negative *A. wagneri* also were obtained from meadow voles (*Microtus pennsylvanicus*) and hispid pocket mice (*Chaetodipus hispidus*; Table 2); *Y. pestis* positive *A. wagneri* were collected from 2 grassland sites and 2 prairie dog colonies.

Individuals from only 3 other flea species were *Y. pestis* positive. A total of 42 *Ctenophthalmus pseudagyrtes* were collected in 2005 from grassland sites 4B and SG (none were collected from any sites in 2004) and only from prairie voles (*Microtus ochrogaster*; Table 1); 20 individual *C. pseudagyrtes* from grassland site SG were *Y. pestis* positive (Table 2). Prairie voles from site SG also harbored the only *Y. pestis* positive individuals (*n* = 23) of *Orchopeas leucopus*, although *Y. pestis* negative individuals of this flea species were also collected from several other sites in both 2004 and 2005 (Table 1) and from multiple rodent species, including deer mice (Table 2). One *Y. pestis* positive *Euhoplopsyllus glacialis* was collected from a desert cottontail rabbit at prairie dog site 5A in 2005, and one *Y. pestis*‐negative individual of this species also was collected via burrow swabbing from prairie dog site CR in 2005 (Tables 1 and 2). Unlike other studies of black‐tailed prairie dogs in Colorado (Salkeld *et al*. 2007; Tripp *et al*. 2009), in this study, *Pulex simulans*, a generalist flea, was very rare and no northern grasshopper mice (*Onychomys leucogaster*) were captured at any sites in either year. Only one individual *P. simulans* was collected in 2004 and none were collected in 2005; the one individual *P. simulans* was *Y. pestis* negative (Table 1).

### Occurrence of plague across sites

The plague status of a site was independent of the plague status of the nearest neighboring site (χ^2^ = 0.219, df = 1, *P* = 0.64). The 8 plague‐positive sites were interspersed among the 6 plague‐negative sites with no apparent correlation between the proximity of plague‐positive and plague‐negative sites (red vs blue symbols in Fig. 1). In addition, the presence of *Y. pestis* at a site was independent of site type (grassland vs prairie dog; χ^2^ = 1.167, df = 1, *P* = 0.28).

### Seroconversion of hosts

In 2004, blood samples collected from 658 individual small mammals (including 217 black‐tailed prairie dogs) trapped at 7 sites were tested for antibodies against the *Y. pestis* F1 antigen; none of the samples were positive. In 2005, blood samples collected from 590 individual small mammals (including 132 black‐tailed prairie dogs) trapped at 14 sites were tested for antibodies against the *Y. pestis* F1 antigen and only 6 (1.0%) were positive. These included 5 deer mice: 2 from grassland site 4B (one titer of 1:128, sampled June 1, 2005; one titer of 1:64, sampled August 17, 2005), 1 from prairie dog site 5A (titer of 1:256, sampled August 16, 2005), and 2 from prairie dog site 4A (one titer of 1:32 and one titer of 1:64, both sampled August 17, 2005); and 1 hispid pocket mouse also from prairie dog site 4A (titer of 1:32, sampled August 17, 2005).

### *Y. pestis* genotyping results

**Figure 3:**
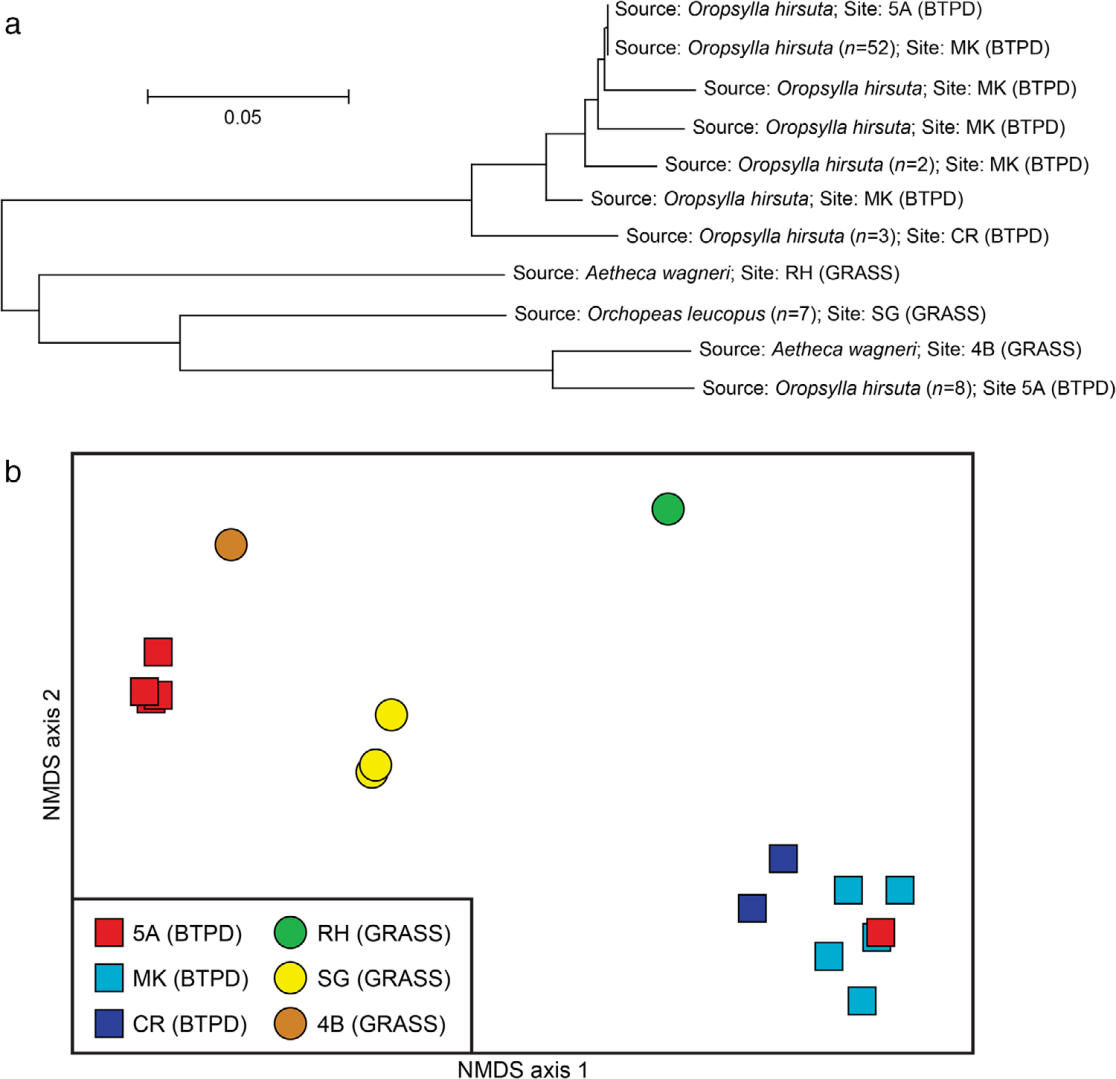
Patterns of population structure in *Y. pestis* that was genotyped from DNA extracted from 78 individual fleas collected in 2005 from 6 sites, including 3 black‐tailed prairie dog colonies (BTPD) and 3 grassland sites (GRASS). (a) Midpoint rooted neighbor‐joining tree created using genetic distance data generated from amplicon size data for 43 *Y*. *pestis* VNTR loci. Length of branches indicates genetic distances between samples. (b) NMDS ordination plot of the same genetic distance data, with squares indicating black‐tailed prairie dog colonies, circles indicating grassland sites, and different colors representing different sites.

Of the 294 fleas identified to be *Y. pestis* positive (Table S1, Supporting Information), 78 (26.5%) of the DNA extracts obtained from those fleas contained sufficient *Y. pestis* DNA to support MLVA genotyping; the resulting data were utilized to construct a neighbor‐joining phylogeny and an NMDS ordination (Fig. 3). A Mantel test indicated that genetic distances among the *Y. pestis* genotypes present in the 78 examined fleas were not correlated with geographic distances corresponding to the locations where those fleas were collected (*R* = −0.041, *P* = 0.35).

### Specificity of published nested PCR assay

The nested PCR assay described by Hanson *et al*. (2007), which targets a portion of the *pla* gene, is neither specific nor sensitive for *Y. pestis*. The external primer set yielded perfect matches to sequences in 16 *Escherichia coli* genomes, 1 *Amphibacillus jilinensis* genome, and 1 *Citrobacter koseri* genome but only 260 *Y. pestis* genomes. The internal primer set yielded perfect matches to sequences in 1 *Amphibacillus jilinensis* genome and 320 *Y. pestis* genomes.

## DISCUSSION

The concept of enzootic plague persisting for multiple years within prairie dog populations was, to our knowledge, first proposed by Cully and Williams (2001). Importantly, they developed this concept only for the white‐tailed prairie dog (*C. leucopus*), which, individually, are highly susceptible to plague, similar to black‐tailed and Gunnison's prairie dogs. However, white‐tailed prairie dogs are the least social species of prairie dog and, as a result, densities within their colonies are much lower than that of black‐tailed and Gunnison's prairie dogs, which likely reduces plague transmission and allows recruitment of new individuals to be greater than mortality due to plague, thereby allowing plague to persist within white‐tailed prairie dog populations for long periods of time (Cully 1989; Cully & Williams 2001). Indeed, plague activity in white‐tailed prairie dog populations can last multiple years, proceed slowly through the overall population, and rarely results in extirpation of individual colonies (Ubico *et al*. 1988; Menkens 1991), which is consistent with the concept of enzootic plague. In contrast, plague outbreaks in black‐tailed and Gunnison's prairie dogs are typically characterized by rapid epizootics that often result in 95–100% mortality in affected colonies (e.g. Ecke & Johnson 1952; Lechleitner *et al*. 1968; Barnes 1993; Cully & Williams 2001; Girard *et al*. 2004). However, despite these well‐documented epizootics, it has recently been proposed that enzootic plague can also occur within black‐tailed prairie dog colonies (Hanson *et al*. 2007; Biggins *et al*. 2010; Matchett *et al*. 2010; Salkeld *et al*. 2010; Mize & Britten 2016; Maestas & Britten 2019; Eads *et al*. 2020), defined by Matchett *et al*. (2010) as “presence of disease‐causing *Y. pestis* without any noticeable decrease in prairie dog abundance.”

In this study, we found no evidence that *Y. pestis* was present—even at low levels—at either black‐tailed prairie dog or grassland sites prior to the widespread plague activity that occurred starting in June of 2005. At 9 of our 14 study sites, we collected and analyzed mammals and fleas in both 2004 and 2005 (Fig. 1; Table 1). Despite collecting and analyzing almost twice as many fleas in 2004 as we did in 2005 (Table 1), none of the fleas that we collected in 2004 were *Y. pestis* positive and none of the 217 black‐tailed prairie dogs (nor any individuals of any of the other examined mammal species) tested in that same year were seropositive. In addition, many of these same sites were also sampled in 2003 (Ray & Collinge 2006) but no *Y. pestis* positive fleas were collected in that year either. However, in 2005, we collected hundreds of *Y. pestis* positive fleas (>25% of the total) from 4 of the sites (grassland site 4B; prairie dog sites 1A, 4A, 5A) that also had been sampled in 2004, as well as 4 other sites that were only sampled in 2005 (grassland sites RH, SG; prairie dog sites CR, MK; Fig. 2; Table 1). In addition, once detected, *Y. pestis* definitely noticeably decreased prairie dog abundance—populations of black‐tailed prairie dogs in the 5 colonies from which we collected *Y. pestis* positive fleas in 2005 (Table 1) were reduced 92–100% following the plague epizootics that occurred within them. Thus, our findings do not support the idea that enzootic plague is occurring among populations of black‐tailed prairie dogs. Rather, they are consistent with the results from multiple other studies that collected fleas from the same black‐tailed prairie dog colonies across several years and, importantly, also tested the collected fleas for the presence of *Y. pestis*, which also found no *Y. pestis* positive fleas in years preceding a plague outbreak (Thiagarajan *et al*. 2008; Stapp *et al*. 2009; Biggins *et al*. 2010; Bron *et al*. 2019), or no *Y. pestis* positive fleas at all if plague outbreaks did not occur during any years of the study (Holmes *et al*. 2006); but also see Romain *et al*. (2013). Of note, once *Y. pestis* positive fleas were detected from black‐tailed prairie dog colonies in the aforementioned studies, plague epizootics followed and resulted in large reductions in the abundance of black‐tailed prairie dogs at all of the affected colonies.

What then is the evidence for enzootic plague among black‐tailed prairie dogs (i.e. the purported long‐term persistence of *Y. pestis* within black‐tailed prairie dog colonies without noticeable reductions in the abundance of black‐tailed prairie dogs)? A modeling study (Salkeld *et al*. 2010) suggested that plague could persist within black‐tailed prairie dog colonies for prolonged periods by moving between prairie dog family groups, but this was not based upon actual testing of fleas for the presence of *Y. pestis*. Several studies (Biggins *et al*. 2010; Eads *et al*. 2020) have examined the effects of treatment of burrows in black‐tailed prairie dog colonies with deltamethrin dust and determined that compared to control colonies, those treatments reduced the abundance of fleas on captured black‐tailed prairie dogs, in their burrows, and/or on other mammal species present in the treated colonies. One of these studies (Biggins *et al*. 2010) suggested that these treatments increased survival of black‐tailed prairie dogs on the treated colonies by suppressing enzootic plague but *Y. pestis* positive fleas collected from black‐tailed prairie dogs were detected only in the examined colonies during active epizootics, not when the colonies were fully active. In addition, increased survival of black‐tailed prairie dogs in colonies treated with insecticides is not definitive proof that enzootic plague was active in those colonies prior to treatment. These treatments drastically reduce the number of fleas present in treated burrows (Tripp *et al*. 2017; Eads *et al*. 2020) but also kill other arthropods present within the treated burrows that could serve as vectors for other infectious diseases that could also cause mortality in prairie dogs (Salkeld *et al*. 2016); removing external parasites would also, on its own, be expected to improve the overall health of prairie dogs. In addition, reducing the number of fleas present in a colony could be preventing *Y. pestis* from being transmitted within a treated colony once introduced from outside the colony.

To our knowledge, the only studies that have detected purported *Y. pestis* positive fleas from normally active black‐tailed prairie dog colonies that did not subsequently undergo plague epizootics have utilized a nested PCR assay targeting a 110 bp region of the *pla* gene of *Y. pestis* that was developed by Hanson *et al*. (2007) by modifying an existing and commonly used non‐nested PCR assay targeting a larger region of the *pla* gene (Hinnebusch & Schwan 1993). This nested PCR assay was first used to examine fleas collected from 48 and 42 active black‐tailed prairie dog colonies in Montana in 2002 and 2003, respectively, and identified purported *Y. pestis* positive fleas from a majority of them (63% in 2002, 57% in 2003); none of the examined colonies were reported to undergo subsequent epizootics. Based upon this finding, Hanson *et al*. (2007) suggested for the first time that black‐tailed prairie dogs could serve as enzootic hosts for plague. Matchett *et al*. (2010) collected hundreds of fleas from black‐footed ferrets (*Mustela nigripes*) and carnivores within active black‐tailed prairie dog colonies in Montana from 1996–2007 and tested them for the presence of *Y. pestis*. No *Y. pestis* positive fleas were detected in that study from 1996–2005 when mouse inoculation and a different PCR assay (Engelthaler *et al*. 1999) were utilized to test for the presence of *Y. pestis* in the collected fleas, but *Y. pestis* was purportedly detected from fleas collected from almost 10% of healthy ferrets sampled in 2006 and 2007 after they started utilizing the nested PCR assay developed by Hanson *et al*. (2007); the black‐tailed prairie dog colonies from which these fleas were collected did not subsequently undergo plague epizootics, so it was concluded that enzootic plague was active in these colonies. Using this same assay, Mize and Britten (2016) identified purported *Y. pestis* positive fleas collected from 13.9% of the black‐tailed prairie dog burrows sampled from active colonies at 5 locations outside the known distribution of plague in the United States where plague had never previously been documented and concluded that enzootic plague was present in these colonies. Most recently, Maestas and Britten (2019) utilized this assay to examine fleas collected from 16 sites in South Dakota, including multiple black‐tailed prairie dog colonies; none of these sites were reported to be experiencing plague epizootics at the time the fleas were collected. They detected 19 purported *Y. pestis* positive fleas collected from multiple sites, including active black‐tailed prairie dog colonies and control sites without prairie dogs. [Correction added on June 14, 2021 after first online publication: The 2 sentences prior to this statement have been amended. The original text read, ‘Most recently, Maestas and Britten (2019) utilized this assay to examine fleas collected from 20 sites in South Dakota, including multiple black‐tailed prairie dog colonies. They detected 19 purported *Y. pestis* positive fleas from 19 of the sites (i.e. one purported *Y. pestis* positive flea per site), including 16 active black‐tailed prairie dog colonies and 2 control sites without prairie dogs, and concluded that enzootic plague was present at these sites.’] The atypical results obtained with this nested PCR assay suggest that it may be yielding false positives results, which is a known potential problem with nested PCR assays (Bretagne 2003; Hayden *et al*. 2004). Indeed, our *in silico* analysis confirmed that this assay is capable of producing both false negative and false positive results, and several other studies have documented that the *pla* gene is not specific to *Y. pestis* (Janse *et al*. 2013; Hansch *et al*. 2015; Giles *et al*. 2016) and, as such, should only be used for detection of *Y. pestis* in concert with other targets (Demeure *et al*. 2019). Given this, we feel that the concept of enzootic plague persisting long‐term within black‐tailed prairie dog populations without a noticeable decrease in the abundance of prairie dogs within those colonies should be considered with caution until these types of findings can be validated with complimentary approaches, such as other *Y. pestis*‐specific PCR assays targeting additional regions of the *Y. pestis* genome (Mitchell *et al*. 2017; Bron *et al*. 2019; Bai *et al*. 2020), mouse inoculation, genotyping of *Y. pestis* present in infected fleas (Girard *et al*. 2004; this study), and/or culture of *Y. pestis* directly from infected fleas (Sarovich *et al*. 2010).

The 2005 plague outbreak within our study area appeared to originate in other mammal species and then spread from those other species into black‐tailed prairie dogs. Although not continuous, our flea collection data document plague activity first at multiple grassland sites before subsequently occurring in other mammals in black‐tailed prairie dog colonies and then in prairie dogs in those same colonies. The first date that *Y. pestis* positive fleas were detected in 2005 occurred earlier at all 3 *Y. pestis* positive grassland sites (June 1–21) than at the 5 *Y. pestis* positive prairie dog colonies (first date June 27; Fig. 2). This was the case even after accounting for the range of collection dates at both types of sites (Fig. 2). Indeed, flea sampling at 2 prairie dog colonies started on May 30, 2005 (sites 4A and 5A), well before sampling began at plague‐positive grassland sites (RH and SG). In addition, at 3 of the 5 *Y. pestis* positive prairie dog colonies (1A, 5A, and MK), *Y. pestis* positive fleas were first reported from other small mammal species also present in these prairie dog colonies prior to *Y. pestis* positive fleas occurring on black‐tailed prairie dogs (Fig. 2). This is additional evidence that *Y. pestis* likely was not already circulating among black‐tailed prairie dogs as enzootic plague prior to the onset of epizootics in black‐tailed prairie dog sites but, rather, was likely introduced from outside those colonies. Indeed, *Y. pestis* may have been introduced from a source or sources completely outside of our study area as we did not detect it in any fleas at any of the sites—both black‐tailed prairie dog colonies and grassland sites—that we sampled in 2004, nor did we detect it at these and other nearby sites in recent previous years (Collinge & Ray 2006).

Our genotyping of *Y. pestis* in infected fleas revealed likely dispersal of *Y. pestis* between some of our study sites and possible multiple introductions of *Y. pestis* to our study area from unknown reservoirs. In general, multiple *Y. pestis* positive fleas collected from the same site yielded highly similar *Y. pestis* genotypes, with one notable exception. The *Y. pestis* present in one prairie dog flea (*O. hirsuta*) collected from prairie dog colony 5A was much more similar to *Y. pestis* present in multiple *O. hirsuta* collected in prairie dog colony MK than it was to *Y. pestis* present in other *O. hirsuta* collected from prairie dog colony 5A (Fig. 3). This suggests movement of *Y. pestis* positive *O. hirsuta* from prairie dog colony MK to prairie dog colony 5A, perhaps by a dispersing black‐tailed prairie dog (Stapp *et al*. 2004); dispersal of black‐tailed prairie dogs between colonies in this area is common (Sackett *et al*. 2012) and these colonies are <10 km apart and, therefore, within the dispersal capability of black‐tailed prairie dogs (Knowles 1985), but it also could have been dispersed via other mechanisms. Coyotes and other highly mobile mammals are known to disperse rodent fleas, especially during plague epizootics (Lechleitner*et al*. 1968; Holmes *et al*. 2006; Salkeld *et al*. 2007; Snall *et al*. 2008; Stapp *et al*. 2009; Jones & Britten 2010; Savage *et al*. 2011). *Y. pestis* present in multiple *O. hirsuta* collected from prairie dog colonies MK and CR were similar (Fig. 3), suggesting *Y. pestis* may have been transferred between these 2 colonies or was introduced to these 2 colonies from a common source. Likewise, *Y. pestis* present in fleas collected from grassland site 4B and prairie dog colony 5A were also similar (Fig. 3). This pattern and the finding that *Y. pestis* positive fleas were first collected from 4B almost 2 months before *Y. pestis* positive fleas were first collected from 5A (Fig. 2) suggest that grassland site 4B may have been a source for the plague epizootic in prairie dog colony 5A. In contrast, *Y. pestis* genotypes from each of the 3 grassland sites were distinct from each other, as were *Y. pestis* genotypes collected from different prairie dog colonies (e.g. 5A vs MK/CR; Fig. 3). Thus, the occurrence of *Y. pestis* in this system was complex and did not follow a pattern of isolation‐by‐distance, as evidenced by plague‐positive sites and plague‐negative sites interspersed together throughout the study area (Fig. 1). This pattern, along with the strong genetic structure we observed within *Y. pestis*, suggests that multiple *Y. pestis* lineages may have been independently introduced to our study area as opposed to a single introduction that then subsequently swept across the entire landscape. As other studies have noted, the most likely explanation for the pattern we observed is a favorable environmental cue that led to the simultaneous amplification of multiple *Y. pestis* lineages, with each dispersing from unknown cryptic sources (Girard *et al*. 2004; Snall *et al*. 2008; Savage*et al*. 2011). It is important to note that this study is a snapshot of *Y. pestis* population structure in the study area and that sampling constraints may have introduced some biases. For example, we did not find a grassland site with similar genotypes to the southernmost prairie dog colony at CR. This does not mean this *Y. pestis* lineage was not present elsewhere on the landscape; it may have been present but not sampled. In addition, we did not analyze fleas from larger mammals, so their possible role in the plague activity in our study area is unknown.

Although we did not identify any mammal species that may serve as long‐term reservoirs for *Y. pestis*, we found evidence that deer mice may have served as a bridging host for introducing *Y. pestis* into black‐tailed prairie dogs. This was possible because we searched for *Y. pestis* in fleas prior to the occurrence of epizootics in black‐tailed prairie dogs, thereby allowing us to overcome a bias inherent in many studies of plague ecology that are only initiated after epizootics begin or end and, as a result, cannot identify species that may be responsible for the initial introduction of plague to an area (Salkeld *et al*. 2016). In both 2004 and 2005, deer mice were the most widespread mammal species in our study area, occurring at 11 of the 14 sites (Table 1), which provided continuity across our study area that could be conducive to plague transmission (Thiagarajan *et al*. 2008). In 2005, *Y. pestis* positive fleas were collected from deer mice at multiple prairie dog colonies and grassland sites and 5 individual deer mice were found to be seropositive, but in 2004, no *Y. pestis* positive fleas were collected from deer mice and no individuals of this species were seropositive, which suggest, as others researchers have noted (Salkeld & Stapp 2008; Eisen & Gage 2009; Danforth *et al*. 2018), that this species likely is not a long‐term reservoir for *Y. pestis*. However, deer mice may have served as a bridging host (Caron *et al*. 2015) for *Y. pestis* between unknown cryptic reservoir hosts that were not sampled in our study and black‐tailed prairie dogs, prior to the onset of epizootic activity in black‐tailed prairie dogs. In 2005, deer mice carried the first *Y. pestis* positive fleas that we collected at 2 of the 3 *Y. pestis* positive grassland sites (4B, RH) and at 2 *Y. pestis* positive prairie dog colonies (1A, MK; Fig. 2) that later experienced widespread plague activity among black‐tailed prairie dogs. Bron *et al*. (2019) also observed a similar pattern of *Y. pestis* positive fleas on deer mice in black‐tailed prairie dog colonies prior to subsequent evidence of plague in prairie dogs and prairie dog fleas in the same colonies. These patterns are compatible with spread of *Y. pestis* from unknown cryptic reservoirs, to deer mice, to black‐tailed prairie dogs; and the transfer of *Y. pestis* from deer mice to prairie dogs in our study area could have been facilitated by *A. wagneri*. Although it is important to note that *A. wagneri* has been documented to be a poor vector of *Y. pestis* (Eskey & Haas 1940; Kartman & Prince 1956; Eisen *et al*. 2008a), as suggested by Eads *et al*. (2020), even very rare transmission of *Y. pestis* by *A. wagneri* may be sufficient for initiating subsequent epizootics in prairie dogs. Although we did not detect any *A. wagneri* on black‐tailed prairie dogs in this study, we did collect *Y. pestis* positive individuals of this flea species from deer mice at multiple prairie dog colonies prior to plague among prairie dogs in those colonies, and other studies have collected this flea species directly from black‐tailed prairie dogs (Eads *et al*. 2020).

Following the plague epizootics that occurred at our *Y. pestis* positive black‐tailed prairie dog sites, deer mice also may have served as spillover hosts. Although host switching by *O. hirsuta* is thought to be rare even after the death of their preferred prairie dog hosts (Salkeld & Stapp 2008; Brinkerhoff *et al*. 2011), Stapp *et al*. (2009) found that *O. hirsuta*, including some positive for *Y. pestis*, were quite common on northern grasshopper mice (*O. leucogaster*) trapped in black‐tailed prairie dog colonies, especially when plague epizootics were occurring in those colonies. We did not trap northern grasshopper mice in our study but we did collect 8 *O. hirsuta* from deer mice (*n* = 4) and dessert cottontail rabbits (*n* = 4; Table 2), suggesting the possibility for interspecific transmission of *Y. pestis* from black‐tailed prairie dogs to deer mice and/or other mammal species. Multiple other studies have suggested that deer mice serve as spillover hosts for *Y. pestis* following epizootics in prairie dogs and other species (Lechleitner *et al*. 1968; Salkeld & Stapp 2008; Salkeld *et al*. 2016; Danforth *et al*. 2018).

*Y. pestis* persists in the environment throughout much of the western United States (Cully & Williams 2001) but how it does that remains a frustrating mystery. Despite significant efforts to do so, no obvious, widespread, long‐term reservoir hosts have been identified (Salkeld *et al*. 2016). One explanation for this is that there is not a single or even several rodent species that are responsible for the long‐term persistence of *Y. pestis* in western North America but, rather, *Y. pestis* is maintained via low‐level transmission among many different mammalian host species and their associated fleas (Gage & Kosoy 2005). This seems plausible given that the western United States has a higher number of potential mammalian hosts for *Y. pestis* than any other region of the world (Mahmoudi *et al*. 2020). Thus, although under certain conditions *Y. pestis* may be maintained locally in a single rodent species (Kosoy *et al*. 2017), in much of the western United States it may be maintained by an almost stochastic pattern of transmission among many different hosts species, which would be extremely difficult to detect and thus study.

In conclusion, we found no evidence that *Y. pestis* was persisting long‐term in the black‐tailed prairie dog colonies that we examined prior to the widespread plague epizootics that drastically reduced prairie dog populations within these colonies, which is not supportive of the idea that enzootic plague occurs within black‐tailed prairie dog populations. In addition, we also found no evidence that *Y. pestis* was persisting in our grassland sites but, rather, was likely introduced from outside our study area to initiate the widespread plague activity that occurred in 2005. *Y. pestis* genotyping results suggest that plague may have been introduced to our study area multiple times from unknown sources and that, once introduced, *Y. pestis* was likely dispersed among some of our study sites. Finally, we identified evidence that deer mice may have served as bridging hosts for *Y. pestis* between cryptic reservoirs and black‐tailed prairie dogs, and as spillover hosts following the subsequent epizootics in black‐tailed prairie dogs.

## Supporting information


**Table S1** 294 *Y. pestis* positive fleas collected in this study
