# Supplementary material for: No evidence for enzootic plague within black‐tailed prairie dog (Cynomys ludovicianus) populations
Source: Integr Zool. 2021 May 31;16(6):834–51. doi: 10.1111/1749-4877.12546 (PMC9292313; doi:10.1111/1749-4877.12546)
Supplement: Supplementary file 1 — Table S1 294 Y. pestis positive fleas collected in this study [file INZ2-16-834-s001.doc]

**SUPPLEMENTARY MATERIALS**

**Table S1** 294 *Y. pestis* positive fleas collected in this study

| **FLEA SPECIES** | **HOST SPECIES** | **SITE** | **SITE TYPE** | **ANALYZED WITH MLVA** | **COLLECTION DATE** |
| --- | --- | --- | --- | --- | --- |
| *Aetheca wagneri* | *Peromyscus maniculatus* | 4B | Grassland | No | 1-Jun-2005 |
| *Aetheca wagneri* | *Peromyscus maniculatus* | 4B | Grassland | Yes | 1-Jun-2005 |
| *Aetheca wagneri* | *Peromyscus maniculatus* | RH | Grassland | No | 9-Jun-2005 |
| *Aetheca wagneri* | *Peromyscus maniculatus* | RH | Grassland | Yes | 9-Jun-2005 |
| *Aetheca wagneri* | *Peromyscus maniculatus* | RH | Grassland | No | 9-Jun-2005 |
| *Ctenophthalmus pseudagyrtes* | *Microtus ochrogaster* | SG | Grassland | No | 21-Jun-2005 |
| *Ctenophthalmus pseudagyrtes* | *Microtus ochrogaster* | SG | Grassland | No | 21-Jun-2005 |
| *Ctenophthalmus pseudagyrtes* | *Microtus ochrogaster* | SG | Grassland | No | 21-Jun-2005 |
| *Ctenophthalmus pseudagyrtes* | *Microtus ochrogaster* | SG | Grassland | No | 21-Jun-2005 |
| *Ctenophthalmus pseudagyrtes* | *Microtus ochrogaster* | SG | Grassland | No | 21-Jun-2005 |
| *Ctenophthalmus pseudagyrtes* | *Microtus ochrogaster* | SG | Grassland | No | 21-Jun-2005 |
| *Ctenophthalmus pseudagyrtes* | *Microtus ochrogaster* | SG | Grassland | No | 21-Jun-2005 |
| *Ctenophthalmus pseudagyrtes* | *Microtus ochrogaster* | SG | Grassland | No | 21-Jun-2005 |
| *Ctenophthalmus pseudagyrtes* | *Microtus ochrogaster* | SG | Grassland | No | 21-Jun-2005 |
| *Ctenophthalmus pseudagyrtes* | *Microtus ochrogaster* | SG | Grassland | No | 21-Jun-2005 |
| *Ctenophthalmus pseudagyrtes* | *Microtus ochrogaster* | SG | Grassland | No | 21-Jun-2005 |
| *Ctenophthalmus pseudagyrtes* | *Microtus ochrogaster* | SG | Grassland | No | 21-Jun-2005 |
| *Ctenophthalmus pseudagyrtes* | *Microtus ochrogaster* | SG | Grassland | No | 21-Jun-2005 |
| *Ctenophthalmus pseudagyrtes* | *Microtus ochrogaster* | SG | Grassland | No | 21-Jun-2005 |
| *Ctenophthalmus pseudagyrtes* | *Microtus ochrogaster* | SG | Grassland | No | 21-Jun-2005 |
| *Ctenophthalmus pseudagyrtes* | *Microtus ochrogaster* | SG | Grassland | No | 21-Jun-2005 |
| *Ctenophthalmus pseudagyrtes* | *Microtus ochrogaster* | SG | Grassland | No | 21-Jun-2005 |
| *Ctenophthalmus pseudagyrtes* | *Microtus ochrogaster* | SG | Grassland | No | 21-Jun-2005 |
| *Ctenophthalmus pseudagyrtes* | *Microtus ochrogaster* | SG | Grassland | No | 21-Jun-2005 |
| *Ctenophthalmus pseudagyrtes* | *Microtus ochrogaster* | SG | Grassland | No | 21-Jun-2005 |
| *Orchopeas leucopus* | *Microtus ochrogaster* | SG | Grassland | No | 21-Jun-2005 |
| *Orchopeas leucopus* | *Microtus ochrogaster* | SG | Grassland | No | 21-Jun-2005 |
| *Orchopeas leucopus* | *Microtus ochrogaster* | SG | Grassland | Yes | 21-Jun-2005 |
| *Orchopeas leucopus* | *Microtus ochrogaster* | SG | Grassland | No | 21-Jun-2005 |
| *Orchopeas leucopus* | *Microtus ochrogaster* | SG | Grassland | Yes | 21-Jun-2005 |
| *Orchopeas leucopus* | *Microtus ochrogaster* | SG | Grassland | Yes | 21-Jun-2005 |
| *Orchopeas leucopus* | *Microtus ochrogaster* | SG | Grassland | Yes | 21-Jun-2005 |
| *Orchopeas leucopus* | *Microtus ochrogaster* | SG | Grassland | No | 21-Jun-2005 |
| *Orchopeas leucopus* | *Microtus ochrogaster* | SG | Grassland | Yes | 21-Jun-2005 |
| *Orchopeas leucopus* | *Microtus ochrogaster* | SG | Grassland | Yes | 21-Jun-2005 |
| *Orchopeas leucopus* | *Microtus ochrogaster* | SG | Grassland | No | 21-Jun-2005 |
| *Orchopeas leucopus* | *Microtus ochrogaster* | SG | Grassland | No | 21-Jun-2005 |
| *Orchopeas leucopus* | *Microtus ochrogaster* | SG | Grassland | No | 21-Jun-2005 |
| *Orchopeas leucopus* | *Microtus ochrogaster* | SG | Grassland | No | 21-Jun-2005 |
| *Orchopeas leucopus* | *Microtus ochrogaster* | SG | Grassland | No | 21-Jun-2005 |
| *Orchopeas leucopus* | *Microtus ochrogaster* | SG | Grassland | No | 21-Jun-2005 |
| *Orchopeas leucopus* | *Microtus ochrogaster* | SG | Grassland | No | 21-Jun-2005 |
| *Orchopeas leucopus* | *Microtus ochrogaster* | SG | Grassland | No | 21-Jun-2005 |
| *Orchopeas leucopus* | *Microtus ochrogaster* | SG | Grassland | No | 21-Jun-2005 |
| *Orchopeas leucopus* | *Microtus ochrogaster* | SG | Grassland | Yes | 21-Jun-2005 |
| *Orchopeas leucopus* | *Microtus ochrogaster* | SG | Grassland | No | 21-Jun-2005 |
| *Orchopeas leucopus* | *Microtus ochrogaster* | SG | Grassland | No | 21-Jun-2005 |
| *Orchopeas leucopus* | *Microtus ochrogaster* | SG | Grassland | No | 21-Jun-2005 |
| *Oropsylla hirsuta* | *Cynomys ludovicianus* | 1A | Black-tailed prairie dog | No | 27-Jun-2005 |
| *Euhoplopsyllus glacialis* | *Sylvilagus audobonii* | 5A | Black-tailed prairie dog | No | 26-Jul-2005 |
| *Oropsylla hirsuta* | *Cynomys ludovicianus* | 5A | Black-tailed prairie dog | No | 27-Jul-2005 |
| *Oropsylla hirsuta* | *Cynomys ludovicianus* | 5A | Black-tailed prairie dog | No | 27-Jul-2005 |
| *Oropsylla hirsuta* | *Cynomys ludovicianus* | 5A | Black-tailed prairie dog | No | 27-Jul-2005 |
| *Oropsylla hirsuta* | *Cynomys ludovicianus* | 5A | Black-tailed prairie dog | No | 27-Jul-2005 |
| *Oropsylla hirsuta* | *Cynomys ludovicianus* | 5A | Black-tailed prairie dog | No | 27-Jul-2005 |
| *Oropsylla hirsuta* | *Cynomys ludovicianus* | 5A | Black-tailed prairie dog | Yes | 27-Jul-2005 |
| *Oropsylla hirsuta* | *Cynomys ludovicianus* | 5A | Black-tailed prairie dog | Yes | 27-Jul-2005 |
| *Oropsylla hirsuta* | *Cynomys ludovicianus* | 5A | Black-tailed prairie dog | Yes | 27-Jul-2005 |
| *Oropsylla hirsuta* | *Cynomys ludovicianus* | 5A | Black-tailed prairie dog | No | 27-Jul-2005 |
| *Oropsylla hirsuta* | *Cynomys ludovicianus* | 5A | Black-tailed prairie dog | Yes | 27-Jul-2005 |
| *Oropsylla hirsuta* | *Cynomys ludovicianus* | 5A | Black-tailed prairie dog | Yes | 27-Jul-2005 |
| *Oropsylla hirsuta* | *Cynomys ludovicianus* | 5A | Black-tailed prairie dog | Yes | 27-Jul-2005 |
| *Oropsylla hirsuta* | *Cynomys ludovicianus* | 4A | Black-tailed prairie dog | No | 28-Jul-2005 |
| *Oropsylla hirsuta* | *Cynomys ludovicianus* | 5A | Black-tailed prairie dog | Yes | 28-Jul-2005 |
| *Oropsylla hirsuta* | *Cynomys ludovicianus* | 5A | Black-tailed prairie dog | Yes | 28-Jul-2005 |
| *Oropsylla hirsuta* | *Cynomys ludovicianus* | 5A | Black-tailed prairie dog | No | 28-Jul-2005 |
| *Oropsylla hirsuta* | *Cynomys ludovicianus* | 5A | Black-tailed prairie dog | No | 28-Jul-2005 |
| *Oropsylla hirsuta* | *Cynomys ludovicianus* | 5A | Black-tailed prairie dog | No | 28-Jul-2005 |
| *Oropsylla hirsuta* | *Cynomys ludovicianus* | 5A | Black-tailed prairie dog | Yes | 29-Jul-2005 |
| *Oropsylla hirsuta* | *Cynomys ludovicianus* | 5A | Black-tailed prairie dog | No | 29-Jul-2005 |
| *Aetheca wagneri* | *Peromyscus maniculatus* | MK | Black-tailed prairie dog | No | 24-Aug-2005 |
| *Oropsylla hirsuta* | *Cynomys ludovicianus* | MK | Black-tailed prairie dog | Yes | 30-Aug-2005 |
| *Oropsylla hirsuta* | *Cynomys ludovicianus* | MK | Black-tailed prairie dog | No | 30-Aug-2005 |
| *Oropsylla hirsuta* | *Cynomys ludovicianus* | MK | Black-tailed prairie dog | No | 30-Aug-2005 |
| *Oropsylla hirsuta* | *Cynomys ludovicianus* | MK | Black-tailed prairie dog | No | 30-Aug-2005 |
| *Oropsylla hirsuta* | *Cynomys ludovicianus* | MK | Black-tailed prairie dog | Yes | 30-Aug-2005 |
| *Oropsylla hirsuta* | *Cynomys ludovicianus* | MK | Black-tailed prairie dog | Yes | 30-Aug-2005 |
| *Oropsylla hirsuta* | *Cynomys ludovicianus* | MK | Black-tailed prairie dog | No | 30-Aug-2005 |
| *Oropsylla hirsuta* | *Cynomys ludovicianus* | MK | Black-tailed prairie dog | No | 30-Aug-2005 |
| *Oropsylla hirsuta* | *Cynomys ludovicianus* | MK | Black-tailed prairie dog | Yes | 30-Aug-2005 |
| *Oropsylla hirsuta* | *Cynomys ludovicianus* | MK | Black-tailed prairie dog | Yes | 30-Aug-2005 |
| *Oropsylla hirsuta* | *Cynomys ludovicianus* | MK | Black-tailed prairie dog | Yes | 30-Aug-2005 |
| *Oropsylla hirsuta* | *Cynomys ludovicianus* | MK | Black-tailed prairie dog | Yes | 30-Aug-2005 |
| *Oropsylla hirsuta* | *Cynomys ludovicianus* | MK | Black-tailed prairie dog | No | 30-Aug-2005 |
| *Oropsylla hirsuta* | *Cynomys ludovicianus* | MK | Black-tailed prairie dog | Yes | 30-Aug-2005 |
| *Oropsylla hirsuta* | *Cynomys ludovicianus* | MK | Black-tailed prairie dog | Yes | 30-Aug-2005 |
| *Oropsylla hirsuta* | *Cynomys ludovicianus* | MK | Black-tailed prairie dog | Yes | 30-Aug-2005 |
| *Oropsylla hirsuta* | *Cynomys ludovicianus* | MK | Black-tailed prairie dog | Yes | 30-Aug-2005 |
| *Oropsylla hirsuta* | *Cynomys ludovicianus* | MK | Black-tailed prairie dog | No | 30-Aug-2005 |
| *Oropsylla hirsuta* | *Cynomys ludovicianus* | MK | Black-tailed prairie dog | Yes | 30-Aug-2005 |
| *Oropsylla hirsuta* | *Cynomys ludovicianus* | MK | Black-tailed prairie dog | Yes | 30-Aug-2005 |
| *Oropsylla hirsuta* | *Cynomys ludovicianus* | MK | Black-tailed prairie dog | No | 30-Aug-2005 |
| *Oropsylla hirsuta* | *Cynomys ludovicianus* | MK | Black-tailed prairie dog | Yes | 30-Aug-2005 |
| *Oropsylla hirsuta* | *Cynomys ludovicianus* | MK | Black-tailed prairie dog | No | 30-Aug-2005 |
| *Oropsylla hirsuta* | *Cynomys ludovicianus* | MK | Black-tailed prairie dog | Yes | 30-Aug-2005 |
| *Oropsylla hirsuta* | *Cynomys ludovicianus* | MK | Black-tailed prairie dog | No | 30-Aug-2005 |
| *Oropsylla hirsuta* | *Cynomys ludovicianus* | MK | Black-tailed prairie dog | No | 30-Aug-2005 |
| *Oropsylla hirsuta* | *Cynomys ludovicianus* | MK | Black-tailed prairie dog | Yes | 30-Aug-2005 |
| *Oropsylla hirsuta* | *Cynomys ludovicianus* | MK | Black-tailed prairie dog | No | 30-Aug-2005 |
| *Oropsylla hirsuta* | *Cynomys ludovicianus* | MK | Black-tailed prairie dog | Yes | 30-Aug-2005 |
| *Oropsylla hirsuta* | *Cynomys ludovicianus* | MK | Black-tailed prairie dog | Yes | 30-Aug-2005 |
| *Oropsylla hirsuta* | *Cynomys ludovicianus* | MK | Black-tailed prairie dog | No | 30-Aug-2005 |
| *Oropsylla hirsuta* | *Cynomys ludovicianus* | MK | Black-tailed prairie dog | Yes | 30-Aug-2005 |
| *Oropsylla hirsuta* | *Cynomys ludovicianus* | MK | Black-tailed prairie dog | No | 30-Aug-2005 |
| *Oropsylla hirsuta* | *Cynomys ludovicianus* | MK | Black-tailed prairie dog | No | 30-Aug-2005 |
| *Oropsylla hirsuta* | *Cynomys ludovicianus* | MK | Black-tailed prairie dog | No | 30-Aug-2005 |
| *Oropsylla hirsuta* | *Cynomys ludovicianus* | MK | Black-tailed prairie dog | Yes | 30-Aug-2005 |
| *Oropsylla hirsuta* | *Cynomys ludovicianus* | MK | Black-tailed prairie dog | Yes | 30-Aug-2005 |
| *Oropsylla hirsuta* | *Cynomys ludovicianus* | MK | Black-tailed prairie dog | Yes | 30-Aug-2005 |
| *Oropsylla hirsuta* | *Cynomys ludovicianus* | MK | Black-tailed prairie dog | No | 30-Aug-2005 |
| *Oropsylla hirsuta* | *Cynomys ludovicianus* | MK | Black-tailed prairie dog | No | 30-Aug-2005 |
| *Oropsylla hirsuta* | *Cynomys ludovicianus* | MK | Black-tailed prairie dog | Yes | 30-Aug-2005 |
| *Oropsylla hirsuta* | *Cynomys ludovicianus* | MK | Black-tailed prairie dog | No | 30-Aug-2005 |
| *Oropsylla hirsuta* | *Cynomys ludovicianus* | MK | Black-tailed prairie dog | Yes | 30-Aug-2005 |
| *Oropsylla hirsuta* | *Cynomys ludovicianus* | MK | Black-tailed prairie dog | No | 30-Aug-2005 |
| *Oropsylla hirsuta* | *Cynomys ludovicianus* | MK | Black-tailed prairie dog | Yes | 30-Aug-2005 |
| *Oropsylla hirsuta* | *Cynomys ludovicianus* | MK | Black-tailed prairie dog | No | 30-Aug-2005 |
| *Oropsylla hirsuta* | *Cynomys ludovicianus* | MK | Black-tailed prairie dog | Yes | 30-Aug-2005 |
| *Oropsylla hirsuta* | *Cynomys ludovicianus* | MK | Black-tailed prairie dog | Yes | 30-Aug-2005 |
| *Oropsylla hirsuta* | *Cynomys ludovicianus* | MK | Black-tailed prairie dog | No | 30-Aug-2005 |
| *Oropsylla hirsuta* | *Cynomys ludovicianus* | MK | Black-tailed prairie dog | Yes | 30-Aug-2005 |
| *Oropsylla hirsuta* | *Cynomys ludovicianus* | MK | Black-tailed prairie dog | No | 30-Aug-2005 |
| *Oropsylla hirsuta* | *Cynomys ludovicianus* | MK | Black-tailed prairie dog | No | 30-Aug-2005 |
| *Oropsylla hirsuta* | *Cynomys ludovicianus* | MK | Black-tailed prairie dog | Yes | 30-Aug-2005 |
| *Oropsylla hirsuta* | *Cynomys ludovicianus* | MK | Black-tailed prairie dog | Yes | 30-Aug-2005 |
| *Oropsylla hirsuta* | *Cynomys ludovicianus* | MK | Black-tailed prairie dog | No | 30-Aug-2005 |
| *Oropsylla hirsuta* | *Cynomys ludovicianus* | MK | Black-tailed prairie dog | Yes | 30-Aug-2005 |
| *Oropsylla hirsuta* | *Cynomys ludovicianus* | MK | Black-tailed prairie dog | Yes | 30-Aug-2005 |
| *Oropsylla hirsuta* | *Cynomys ludovicianus* | MK | Black-tailed prairie dog | Yes | 30-Aug-2005 |
| *Oropsylla hirsuta* | *Cynomys ludovicianus* | MK | Black-tailed prairie dog | Yes | 30-Aug-2005 |
| *Oropsylla hirsuta* | *Cynomys ludovicianus* | MK | Black-tailed prairie dog | No | 30-Aug-2005 |
| *Oropsylla hirsuta* | *Cynomys ludovicianus* | MK | Black-tailed prairie dog | No | 30-Aug-2005 |
| *Oropsylla hirsuta* | *Cynomys ludovicianus* | MK | Black-tailed prairie dog | No | 30-Aug-2005 |
| *Oropsylla hirsuta* | *Cynomys ludovicianus* | MK | Black-tailed prairie dog | Yes | 30-Aug-2005 |
| *Oropsylla hirsuta* | *Cynomys ludovicianus* | MK | Black-tailed prairie dog | No | 30-Aug-2005 |
| *Oropsylla hirsuta* | *Cynomys ludovicianus* | MK | Black-tailed prairie dog | No | 30-Aug-2005 |
| *Oropsylla hirsuta* | *Cynomys ludovicianus* | MK | Black-tailed prairie dog | No | 30-Aug-2005 |
| *Oropsylla hirsuta* | *Cynomys ludovicianus* | MK | Black-tailed prairie dog | No | 30-Aug-2005 |
| *Oropsylla hirsuta* | *Cynomys ludovicianus* | MK | Black-tailed prairie dog | No | 30-Aug-2005 |
| *Oropsylla hirsuta* | *Cynomys ludovicianus* | MK | Black-tailed prairie dog | Yes | 30-Aug-2005 |
| *Oropsylla hirsuta* | *Cynomys ludovicianus* | MK | Black-tailed prairie dog | No | 30-Aug-2005 |
| *Oropsylla hirsuta* | *Cynomys ludovicianus* | MK | Black-tailed prairie dog | No | 30-Aug-2005 |
| *Oropsylla hirsuta* | *Cynomys ludovicianus* | MK | Black-tailed prairie dog | No | 30-Aug-2005 |
| *Oropsylla hirsuta* | *Cynomys ludovicianus* | MK | Black-tailed prairie dog | Yes | 30-Aug-2005 |
| *Oropsylla hirsuta* | *Cynomys ludovicianus* | MK | Black-tailed prairie dog | No | 30-Aug-2005 |
| *Oropsylla hirsuta* | *Cynomys ludovicianus* | MK | Black-tailed prairie dog | No | 30-Aug-2005 |
| *Oropsylla hirsuta* | *Cynomys ludovicianus* | MK | Black-tailed prairie dog | Yes | 30-Aug-2005 |
| *Oropsylla hirsuta* | *Cynomys ludovicianus* | MK | Black-tailed prairie dog | Yes | 30-Aug-2005 |
| *Oropsylla hirsuta* | *Cynomys ludovicianus* | MK | Black-tailed prairie dog | Yes | 30-Aug-2005 |
| *Oropsylla hirsuta* | *Cynomys ludovicianus* | MK | Black-tailed prairie dog | Yes | 30-Aug-2005 |
| *Oropsylla hirsuta* | *Cynomys ludovicianus* | MK | Black-tailed prairie dog | No | 30-Aug-2005 |
| *Oropsylla hirsuta* | *Cynomys ludovicianus* | MK | Black-tailed prairie dog | No | 30-Aug-2005 |
| *Oropsylla hirsuta* | *Cynomys ludovicianus* | MK | Black-tailed prairie dog | No | 30-Aug-2005 |
| *Oropsylla hirsuta* | *Cynomys ludovicianus* | MK | Black-tailed prairie dog | No | 30-Aug-2005 |
| *Oropsylla hirsuta* | *Cynomys ludovicianus* | MK | Black-tailed prairie dog | No | 30-Aug-2005 |
| *Oropsylla hirsuta* | *Cynomys ludovicianus* | MK | Black-tailed prairie dog | No | 30-Aug-2005 |
| *Oropsylla hirsuta* | *Cynomys ludovicianus* | MK | Black-tailed prairie dog | Yes | 30-Aug-2005 |
| *Oropsylla hirsuta* | *Cynomys ludovicianus* | MK | Black-tailed prairie dog | No | 30-Aug-2005 |
| *Oropsylla hirsuta* | *Cynomys ludovicianus* | MK | Black-tailed prairie dog | No | 30-Aug-2005 |
| *Oropsylla hirsuta* | *Cynomys ludovicianus* | MK | Black-tailed prairie dog | Yes | 30-Aug-2005 |
| *Oropsylla hirsuta* | *Cynomys ludovicianus* | MK | Black-tailed prairie dog | No | 30-Aug-2005 |
| *Oropsylla hirsuta* | *Cynomys ludovicianus* | MK | Black-tailed prairie dog | No | 30-Aug-2005 |
| *Oropsylla hirsuta* | *Cynomys ludovicianus* | MK | Black-tailed prairie dog | No | 30-Aug-2005 |
| *Oropsylla hirsuta* | *Cynomys ludovicianus* | MK | Black-tailed prairie dog | No | 30-Aug-2005 |
| *Oropsylla hirsuta* | *Cynomys ludovicianus* | MK | Black-tailed prairie dog | No | 30-Aug-2005 |
| *Oropsylla hirsuta* | *Cynomys ludovicianus* | MK | Black-tailed prairie dog | Yes | 30-Aug-2005 |
| *Oropsylla hirsuta* | *Cynomys ludovicianus* | MK | Black-tailed prairie dog | No | 30-Aug-2005 |
| *Oropsylla hirsuta* | *Cynomys ludovicianus* | MK | Black-tailed prairie dog | No | 30-Aug-2005 |
| *Oropsylla hirsuta* | *Cynomys ludovicianus* | MK | Black-tailed prairie dog | No | 30-Aug-2005 |
| *Oropsylla hirsuta* | *Cynomys ludovicianus* | MK | Black-tailed prairie dog | No | 30-Aug-2005 |
| *Oropsylla hirsuta* | *Cynomys ludovicianus* | MK | Black-tailed prairie dog | No | 30-Aug-2005 |
| *Oropsylla hirsuta* | *Cynomys ludovicianus* | MK | Black-tailed prairie dog | No | 30-Aug-2005 |
| *Oropsylla hirsuta* | *Cynomys ludovicianus* | MK | Black-tailed prairie dog | No | 30-Aug-2005 |
| *Oropsylla hirsuta* | *Cynomys ludovicianus* | MK | Black-tailed prairie dog | No | 30-Aug-2005 |
| *Oropsylla hirsuta* | *Cynomys ludovicianus* | MK | Black-tailed prairie dog | Yes | 30-Aug-2005 |
| *Oropsylla hirsuta* | *Cynomys ludovicianus* | MK | Black-tailed prairie dog | No | 30-Aug-2005 |
| *Oropsylla hirsuta* | *Cynomys ludovicianus* | MK | Black-tailed prairie dog | No | 30-Aug-2005 |
| *Oropsylla hirsuta* | *Cynomys ludovicianus* | MK | Black-tailed prairie dog | No | 30-Aug-2005 |
| *Oropsylla hirsuta* | *Cynomys ludovicianus* | MK | Black-tailed prairie dog | No | 30-Aug-2005 |
| *Oropsylla hirsuta* | *Cynomys ludovicianus* | MK | Black-tailed prairie dog | No | 30-Aug-2005 |
| *Oropsylla hirsuta* | *Cynomys ludovicianus* | MK | Black-tailed prairie dog | No | 30-Aug-2005 |
| *Oropsylla hirsuta* | *Cynomys ludovicianus* | MK | Black-tailed prairie dog | No | 30-Aug-2005 |
| *Oropsylla hirsuta* | *Cynomys ludovicianus* | MK | Black-tailed prairie dog | No | 30-Aug-2005 |
| *Oropsylla hirsuta* | *Cynomys ludovicianus* | MK | Black-tailed prairie dog | No | 30-Aug-2005 |
| *Oropsylla hirsuta* | *Cynomys ludovicianus* | MK | Black-tailed prairie dog | No | 30-Aug-2005 |
| *Oropsylla hirsuta* | *Cynomys ludovicianus* | MK | Black-tailed prairie dog | Yes | 30-Aug-2005 |
| *Oropsylla hirsuta* | *Cynomys ludovicianus* | MK | Black-tailed prairie dog | No | 30-Aug-2005 |
| *Oropsylla hirsuta* | *Cynomys ludovicianus* | MK | Black-tailed prairie dog | No | 30-Aug-2005 |
| *Oropsylla hirsuta* | *Cynomys ludovicianus* | MK | Black-tailed prairie dog | No | 30-Aug-2005 |
| *Oropsylla hirsuta* | *Cynomys ludovicianus* | MK | Black-tailed prairie dog | No | 30-Aug-2005 |
| *Oropsylla hirsuta* | *Cynomys ludovicianus* | MK | Black-tailed prairie dog | Yes | 30-Aug-2005 |
| *Oropsylla hirsuta* | *Cynomys ludovicianus* | MK | Black-tailed prairie dog | No | 30-Aug-2005 |
| *Oropsylla hirsuta* | *Cynomys ludovicianus* | MK | Black-tailed prairie dog | No | 30-Aug-2005 |
| *Oropsylla hirsuta* | *Cynomys ludovicianus* | MK | Black-tailed prairie dog | No | 30-Aug-2005 |
| *Oropsylla hirsuta* | *Cynomys ludovicianus* | MK | Black-tailed prairie dog | No | 30-Aug-2005 |
| *Oropsylla hirsuta* | *Cynomys ludovicianus* | MK | Black-tailed prairie dog | No | 30-Aug-2005 |
| *Oropsylla hirsuta* | *Cynomys ludovicianus* | MK | Black-tailed prairie dog | No | 30-Aug-2005 |
| *Oropsylla hirsuta* | *Cynomys ludovicianus* | MK | Black-tailed prairie dog | Yes | 30-Aug-2005 |
| *Oropsylla hirsuta* | *Cynomys ludovicianus* | MK | Black-tailed prairie dog | No | 30-Aug-2005 |
| *Oropsylla hirsuta* | *Cynomys ludovicianus* | MK | Black-tailed prairie dog | Yes | 30-Aug-2005 |
| *Oropsylla hirsuta* | *Cynomys ludovicianus* | MK | Black-tailed prairie dog | Yes | 30-Aug-2005 |
| *Oropsylla hirsuta* | *Cynomys ludovicianus* | MK | Black-tailed prairie dog | No | 30-Aug-2005 |
| *Oropsylla hirsuta* | *Cynomys ludovicianus* | MK | Black-tailed prairie dog | No | 30-Aug-2005 |
| *Oropsylla hirsuta* | *Cynomys ludovicianus* | MK | Black-tailed prairie dog | No | 30-Aug-2005 |
| *Oropsylla hirsuta* | *Cynomys ludovicianus* | MK | Black-tailed prairie dog | No | 30-Aug-2005 |
| *Oropsylla hirsuta* | *Cynomys ludovicianus* | MK | Black-tailed prairie dog | No | 30-Aug-2005 |
| *Oropsylla hirsuta* | *Cynomys ludovicianus* | MK | Black-tailed prairie dog | No | 30-Aug-2005 |
| *Oropsylla hirsuta* | *Cynomys ludovicianus* | MK | Black-tailed prairie dog | No | 30-Aug-2005 |
| *Oropsylla hirsuta* | *Cynomys ludovicianus* | MK | Black-tailed prairie dog | No | 30-Aug-2005 |
| *Oropsylla hirsuta* | *Cynomys ludovicianus* | MK | Black-tailed prairie dog | No | 30-Aug-2005 |
| *Oropsylla hirsuta* | *Cynomys ludovicianus* | MK | Black-tailed prairie dog | No | 30-Aug-2005 |
| *Oropsylla hirsuta* | *Cynomys ludovicianus* | MK | Black-tailed prairie dog | No | 30-Aug-2005 |
| *Oropsylla hirsuta* | *Cynomys ludovicianus* | MK | Black-tailed prairie dog | No | 30-Aug-2005 |
| *Oropsylla hirsuta* | *Cynomys ludovicianus* | MK | Black-tailed prairie dog | No | 30-Aug-2005 |
| *Oropsylla hirsuta* | *Cynomys ludovicianus* | MK | Black-tailed prairie dog | No | 30-Aug-2005 |
| *Oropsylla hirsuta* | *Cynomys ludovicianus* | MK | Black-tailed prairie dog | No | 30-Aug-2005 |
| *Oropsylla hirsuta* | *Cynomys ludovicianus* | MK | Black-tailed prairie dog | No | 30-Aug-2005 |
| *Oropsylla hirsuta* | *Cynomys ludovicianus* | MK | Black-tailed prairie dog | No | 30-Aug-2005 |
| *Oropsylla hirsuta* | *Cynomys ludovicianus* | MK | Black-tailed prairie dog | Yes | 30-Aug-2005 |
| *Oropsylla hirsuta* | *Cynomys ludovicianus* | MK | Black-tailed prairie dog | No | 30-Aug-2005 |
| *Oropsylla hirsuta* | *Cynomys ludovicianus* | MK | Black-tailed prairie dog | No | 30-Aug-2005 |
| *Oropsylla hirsuta* | *Cynomys ludovicianus* | MK | Black-tailed prairie dog | No | 30-Aug-2005 |
| *Oropsylla hirsuta* | *Cynomys ludovicianus* | MK | Black-tailed prairie dog | No | 30-Aug-2005 |
| *Oropsylla hirsuta* | *Cynomys ludovicianus* | MK | Black-tailed prairie dog | No | 30-Aug-2005 |
| *Oropsylla hirsuta* | *Cynomys ludovicianus* | MK | Black-tailed prairie dog | No | 30-Aug-2005 |
| *Oropsylla hirsuta* | *Cynomys ludovicianus* | MK | Black-tailed prairie dog | No | 30-Aug-2005 |
| *Oropsylla hirsuta* | *Cynomys ludovicianus* | MK | Black-tailed prairie dog | No | 30-Aug-2005 |
| *Oropsylla hirsuta* | *Cynomys ludovicianus* | MK | Black-tailed prairie dog | No | 30-Aug-2005 |
| *Oropsylla hirsuta* | *Cynomys ludovicianus* | MK | Black-tailed prairie dog | No | 30-Aug-2005 |
| *Oropsylla hirsuta* | *Cynomys ludovicianus* | MK | Black-tailed prairie dog | No | 30-Aug-2005 |
| *Oropsylla hirsuta* | *Cynomys ludovicianus* | MK | Black-tailed prairie dog | No | 30-Aug-2005 |
| *Oropsylla hirsuta* | *Cynomys ludovicianus* | MK | Black-tailed prairie dog | No | 30-Aug-2005 |
| *Oropsylla hirsuta* | *Cynomys ludovicianus* | MK | Black-tailed prairie dog | No | 30-Aug-2005 |
| *Oropsylla hirsuta* | *Cynomys ludovicianus* | MK | Black-tailed prairie dog | No | 30-Aug-2005 |
| *Oropsylla hirsuta* | *Cynomys ludovicianus* | MK | Black-tailed prairie dog | No | 30-Aug-2005 |
| *Oropsylla hirsuta* | *Cynomys ludovicianus* | MK | Black-tailed prairie dog | No | 30-Aug-2005 |
| *Oropsylla hirsuta* | *Cynomys ludovicianus* | MK | Black-tailed prairie dog | Yes | 30-Aug-2005 |
| *Oropsylla hirsuta* | *Cynomys ludovicianus* | MK | Black-tailed prairie dog | No | 30-Aug-2005 |
| *Oropsylla hirsuta* | *Cynomys ludovicianus* | MK | Black-tailed prairie dog | No | 30-Aug-2005 |
| *Oropsylla hirsuta* | *Cynomys ludovicianus* | MK | Black-tailed prairie dog | No | 30-Aug-2005 |
| *Oropsylla hirsuta* | *Cynomys ludovicianus* | MK | Black-tailed prairie dog | No | 30-Aug-2005 |
| *Oropsylla hirsuta* | *Cynomys ludovicianus* | MK | Black-tailed prairie dog | No | 30-Aug-2005 |
| *Oropsylla hirsuta* | *Cynomys ludovicianus* | MK | Black-tailed prairie dog | No | 30-Aug-2005 |
| *Oropsylla hirsuta* | *Cynomys ludovicianus* | MK | Black-tailed prairie dog | No | 30-Aug-2005 |
| *Oropsylla hirsuta* | *Cynomys ludovicianus* | MK | Black-tailed prairie dog | No | 30-Aug-2005 |
| *Oropsylla hirsuta* | *Cynomys ludovicianus* | MK | Black-tailed prairie dog | No | 30-Aug-2005 |
| *Oropsylla hirsuta* | *Cynomys ludovicianus* | MK | Black-tailed prairie dog | No | 30-Aug-2005 |
| *Oropsylla hirsuta* | *Cynomys ludovicianus* | MK | Black-tailed prairie dog | No | 30-Aug-2005 |
| *Oropsylla hirsuta* | *Cynomys ludovicianus* | MK | Black-tailed prairie dog | No | 30-Aug-2005 |
| *Oropsylla hirsuta* | *Cynomys ludovicianus* | MK | Black-tailed prairie dog | No | 30-Aug-2005 |
| *Oropsylla hirsuta* | *Cynomys ludovicianus* | MK | Black-tailed prairie dog | No | 30-Aug-2005 |
| *Oropsylla hirsuta* | *Cynomys ludovicianus* | MK | Black-tailed prairie dog | No | 30-Aug-2005 |
| *Oropsylla hirsuta* | *Cynomys ludovicianus* | MK | Black-tailed prairie dog | No | 30-Aug-2005 |
| *Oropsylla hirsuta* | *Cynomys ludovicianus* | MK | Black-tailed prairie dog | No | 30-Aug-2005 |
| *Oropsylla hirsuta* | *Cynomys ludovicianus* | MK | Black-tailed prairie dog | No | 30-Aug-2005 |
| *Oropsylla hirsuta* | *Cynomys ludovicianus* | MK | Black-tailed prairie dog | No | 30-Aug-2005 |
| *Oropsylla hirsuta* | *Cynomys ludovicianus* | MK | Black-tailed prairie dog | Yes | 30-Aug-2005 |
| *Oropsylla hirsuta* | *Cynomys ludovicianus* | MK | Black-tailed prairie dog | No | 30-Aug-2005 |
| *Oropsylla hirsuta* | *Cynomys ludovicianus* | MK | Black-tailed prairie dog | No | 30-Aug-2005 |
| *Oropsylla hirsuta* | *Cynomys ludovicianus* | MK | Black-tailed prairie dog | No | 30-Aug-2005 |
| *Oropsylla hirsuta* | *Cynomys ludovicianus* | MK | Black-tailed prairie dog | No | 30-Aug-2005 |
| *Oropsylla hirsuta* | *Cynomys ludovicianus* | MK | Black-tailed prairie dog | Yes | 30-Aug-2005 |
| *Oropsylla hirsuta* | *Cynomys ludovicianus* | MK | Black-tailed prairie dog | No | 30-Aug-2005 |
| *Oropsylla hirsuta* | *Cynomys ludovicianus* | MK | Black-tailed prairie dog | No | 30-Aug-2005 |
| *Oropsylla hirsuta* | *Cynomys ludovicianus* | MK | Black-tailed prairie dog | No | 30-Aug-2005 |
| *Oropsylla hirsuta* | *Cynomys ludovicianus* | MK | Black-tailed prairie dog | No | 30-Aug-2005 |
| *Oropsylla hirsuta* | *Cynomys ludovicianus* | MK | Black-tailed prairie dog | No | 30-Aug-2005 |
| *Oropsylla hirsuta* | *Cynomys ludovicianus* | MK | Black-tailed prairie dog | No | 30-Aug-2005 |
| *Oropsylla hirsuta* | *Cynomys ludovicianus* | MK | Black-tailed prairie dog | No | 30-Aug-2005 |
| *Oropsylla hirsuta* | *Cynomys ludovicianus* | MK | Black-tailed prairie dog | No | 30-Aug-2005 |
| *Oropsylla hirsuta* | *Cynomys ludovicianus* | MK | Black-tailed prairie dog | Yes | 30-Aug-2005 |
| *Oropsylla hirsuta* | *Cynomys ludovicianus* | MK | Black-tailed prairie dog | No | 30-Aug-2005 |
| *Oropsylla hirsuta* | *Cynomys ludovicianus* | MK | Black-tailed prairie dog | No | 30-Aug-2005 |
| *Oropsylla hirsuta* | *Cynomys ludovicianus* | MK | Black-tailed prairie dog | Yes | 30-Aug-2005 |
| *Oropsylla hirsuta* | *Cynomys ludovicianus* | MK | Black-tailed prairie dog | No | 30-Aug-2005 |
| *Oropsylla hirsuta* | *Cynomys ludovicianus* | MK | Black-tailed prairie dog | No | 30-Aug-2005 |
| *Oropsylla hirsuta* | *Cynomys ludovicianus* | MK | Black-tailed prairie dog | No | 30-Aug-2005 |
| *Oropsylla hirsuta* | *Cynomys ludovicianus* | MK | Black-tailed prairie dog | Yes | 30-Aug-2005 |
| *Oropsylla hirsuta* | *Cynomys ludovicianus* | MK | Black-tailed prairie dog | No | 30-Aug-2005 |
| *Oropsylla hirsuta* | *Cynomys ludovicianus* | MK | Black-tailed prairie dog | No | 30-Aug-2005 |
| *Oropsylla hirsuta* | *Cynomys ludovicianus* | MK | Black-tailed prairie dog | No | 30-Aug-2005 |
| *Aetheca wagneri* | *Peromyscus maniculatus* | MK | Black-tailed prairie dog | No | 31-Aug-2005 |
| *Aetheca wagneri* | *Peromyscus maniculatus* | MK | Black-tailed prairie dog | No | 31-Aug-2005 |
| *Oropsylla hirsuta* | *Peromyscus maniculatus* | MK | Black-tailed prairie dog | No | 31-Aug-2005 |
| *Aetheca wagneri* | *Peromyscus maniculatus* | 1A | Black-tailed prairie dog | No | 2-Sep-2005 |
| *Oropsylla hirsuta* | *Cynomys ludovicianus* | MK | Black-tailed prairie dog | No | 2-Sep-2005 |
| *Oropsylla hirsuta* | NA: Burrow swab | CR | Black-tailed prairie dog | Yes | 28-Sep-2005 |
| *Oropsylla hirsuta* | NA: Burrow swab | CR | Black-tailed prairie dog | Yes | 28-Sep-2005 |
| *Oropsylla hirsuta* | NA: Burrow swab | CR | Black-tailed prairie dog | No | 28-Sep-2005 |
| *Oropsylla hirsuta* | NA: Burrow swab | CR | Black-tailed prairie dog | No | 28-Sep-2005 |
| *Oropsylla hirsuta* | NA: Burrow swab | CR | Black-tailed prairie dog | No | 28-Sep-2005 |
| *Oropsylla hirsuta* | NA: Burrow swab | CR | Black-tailed prairie dog | Yes | 28-Sep-2005 |
| *Oropsylla hirsuta* | NA: Burrow swab | CR | Black-tailed prairie dog | No | 28-Sep-2005 |
| *Oropsylla hirsuta* | NA: Burrow swab | CR | Black-tailed prairie dog | No | 28-Sep-2005 |
